# Polyphotosensitizer‐Based Nanoparticles with Michael Addition Acceptors Inhibiting GST Activity and Cisplatin Deactivation for Enhanced Chemotherapy and Photodynamic Immunotherapy

**DOI:** 10.1002/advs.202300175

**Published:** 2023-03-17

**Authors:** Qinxin Zhao, Ganghao Liang, Boda Guo, Wenkuan Wang, Chao Yang, Dong Chen, Feiya Yang, Haihua Xiao, Nianzeng Xing

**Affiliations:** ^1^ Department of Urology National Cancer Center/National Clinical Research Center for Cancer/Cancer Hospital Chinese Academy of Medical Sciences and Peking Union Medical College Beijing 100021 China; ^2^ State Key Laboratory of Molecular Oncology National Cancer Center/National Clinical Research Center for Cancer/Cancer Hospital Chinese Academy of Medical Sciences and Peking Union Medical College Beijing 100021 China; ^3^ Department of Urology Shanxi Province Cancer Hospital/Shanxi Hospital Affiliated to Cancer Hospital Chinese Academy of Medical Sciences/Cancer Hospital Affiliated to Shanxi Medical University Shanxi 030013 China; ^4^ Beijing National Laboratory for Molecular Sciences Laboratory of Polymer Physics and Chemistry Institute of Chemistry Chinese Academy of Sciences Beijing 100190 China; ^5^ University of Chinese Academy of Sciences Beijing 100049 China

**Keywords:** chemotherapy, immunotherapy, Michael addition acceptor, nanoparticles, photodynamic, prostate cancer

## Abstract

Glutathione S‐transferase (GST), which is a key enzyme in the conjugation reaction of glutathione (GSH), is overexpressed in cancer cells, leading to cisplatin deactivation and ultimately drug resistance. In addition, many tumors are immune “cold tumors,” limiting the application of immune checkpoint inhibitors. Herein, a reactive oxygen species (ROS)‐responsive polyphotosensitizer‐based nanoparticle (NP2) with Michael addition acceptors inhibiting GST activity and cisplatin deactivation is designed. Under the 808 nm light irradiation, on the one hand, the Michael addition acceptor in NP2 can react with GST and inhibit its activity, thereby decreasing the GSH conjugation and reducing the GSH‐mediated deactivation of cisplatin and improving its chemotherapeutic effect. On the other hand, NP2+L induces more ROS production in prostate tumor cells, which can further induce type II immunogenic cell death (ICD) and stimulate a stronger antitumor immune response. It is found that NP2 under the 808 nm light irradiation (NP2+L) can increase PD‐L1 expression on the surface of prostate cancer cells. Subsequently, NP2+L combined with PD‐L1 treatment is found to simultaneously enhance the efficacies of chemotherapy and photodynamic immunotherapy in prostate tumors, providing a new paradigm for the clinical multimodal treatment of tumors.

## Graphical abstract

A novel reactive oxygen species‐responsive polyphotosensitizer‐based nanoparticle (NP2) with Michael addition acceptors inhibiting glutathione S‐transferase activity and cisplatin deactivation is designed. Under the 808 nm light irradiation, NP2 combined with PD‐L1 treatment is found to enhance chemotherapy and photodynamic immunotherapy in prostate tumors simultaneously, providing a new paradigm for clinical multimodal treatment of tumors.

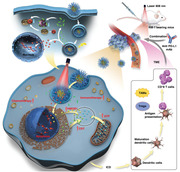

## 1 Introduction

Prostate cancer has high morbidity and mortality in men.^[^ ^1^ ^]^ Chemotherapy is an important treatment for advanced prostate cancers. However, prostate cancer is insensitive to cisplatin, and cisplatin has great systemic side effects. Due to its premature hydrolysis and fast clearance in blood circulation, it has very low bioavailability.^[^ ^2^ ^]^ In addition, an important reason for cisplatin tolerance is the presence of high levels of a detoxifying enzyme, glutathione S‐transferase (GST), in tumor cells.^[^ ^3^ ^]^ GST is a key enzyme involved in the initiation step of the catalytic glutathione (GSH) conjugation reaction. GSH can rapidly bind to cisplatin, leading to cisplatin deactivation and thus reducing the anticancer activity of cisplatin.^[^ ^4^ ^]^ Therefore, enhancing the stability of cisplatin and inhibiting intracellular GST activity are extremely important to reduce the toxic side effects of cisplatin and improve its antitumor efficacy.

In recent years, a class of platinum (IV) prodrugs that can greatly increase the stability of cisplatin has been developed. Platinum (IV) prodrugs can be specifically reduced to platinum (II) complexes by excess intracellular reducing agents in cancer cells to exert their anticancer effects.^[^ ^5^ ^]^ Moreover, many nanodrug delivery systems for platinum (IV) prodrugs have been reported for various tumors.^[^ ^6^, ^7^, ^8^ ^]^ Mechanistically, platinum (IV) prodrugs and their nanodelivery systems can release cisplatin upon entering cancer cells and complete the kill.^[^ ^8^ ^]^ However, as mentioned earlier, these molecules of cisplatin are also readily hydrolyzed and can still react with overexpressed intracellular GSH, leading to its deactivation and further excretion from the cancer cells. Therefore, inhibition of GST activity in tumor cells to reduce the conjugation reaction could be pivotal in enhancing the antitumor activity of platinum (IV) prodrugs and their drug delivery systems.

Immunotherapy has become a hot research topic in the treatment of advanced prostate cancers in recent years.^[^ ^9^ ^]^ However, advanced prostate cancers are “cold tumors” with an immunosuppressive tumor microenvironment (TME), thereby leading to the failure of existing immunotherapeutic regimes.^[^ ^10^ ^]^ In recent years, photodynamic therapy (PDT) that can not only kill cancer cells by activating the photosensitizers to produce reactive species (ROS) under light irradiation, but also trigger the so‐called type II immunogenic cell death (ICD) has been developed.^[^ ^11^ ^]^ Compared with type I ICD, Type II ICD can trigger ER stress directly by PDT.^[^ ^12^ ^]^ Results have shown that tumor cells undergoing type II ICD can release damage‐associated molecular patterns (DMAPs): calreticulin (CRT) exposed on their cell surface, adenosine triphosphate (ATP) secreted, and high mobility‐group protein box 1 (HMGB1) released.^[^ ^13^ ^]^ Recent studies have shown that PDT could promote the apoptosis of tumor cells. Moreover, PDT could promote the uptake and presentation of tumor‐associated antigens by antigen‐presenting cells, reactivate the recognition and attack of tumor cells by T cells, and allow more infiltration of CD8^+^T cells, thereby transforming immune “cold tumors” into immune “hot tumors” and finally improving the responses to immune checkpoint inhibitors.^[^ ^14^ ^]^ In recent years, more studies have shown that multimodal treatment can effectively inhibit tumor growth and produce better outcomes.^[^ ^15^ ^]^ Therefore, PDT combined with chemotherapy and immunotherapy may tackle prostate cancers’ insensitivity to cisplatin. In addition, PDT could also improve the efficacy of combination therapy with immune checkpoint inhibitors.

**Scheme 1:**
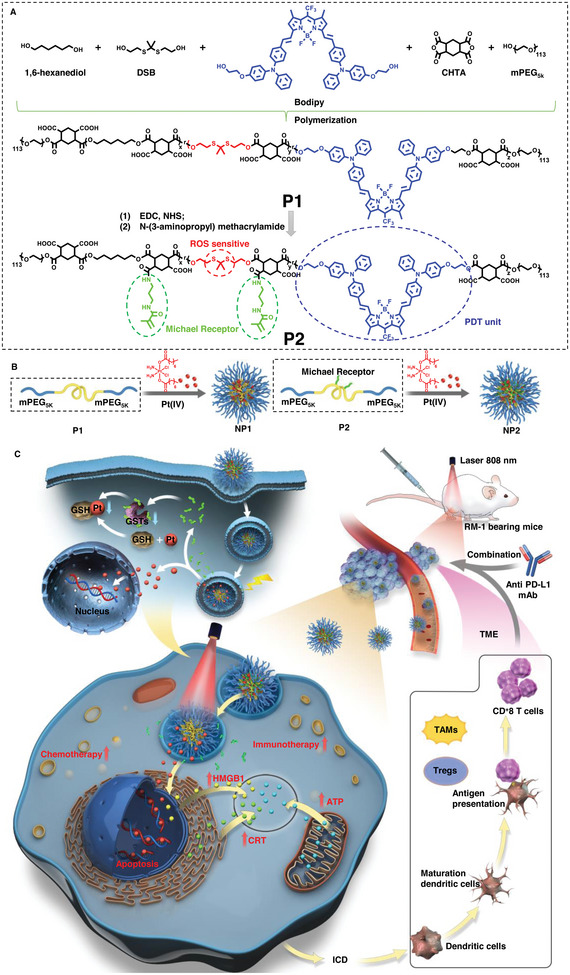
Schematic illustration of NPs for chemotherapy and photodynamic immunotherapy. A) The synthesis of polyphotosensitizer (P1) and subsequent derivatization into P2 with photodynamic Bodipy units and Michael addition acceptors. B) Encapsulation of Pt1 into NP1 and NP2, respectively. C) The possible intracellular fate as well as the mechanism of action of NP2 for chemotherapy and photodynamic immunotherapy. Specifically, NP2 was internalized by prostate cancer cells. Subsequently, on the one hand, NP2 under 808 nm NIR light irradiation (NP2+L) could generate ROS, which could further break the thioketal bonds in P2, resulting in the degradation the of polymer, the dissociation of nanoparticles, as well as the release of cisplatin. In this process, the Michael acceptors could inhibit GSTs, thereby decreasing the intracellular GSH‐mediated cisplatin deactivation. Therefore, the antitumor effect of cisplatin could be enhanced. On the other hand, the ROS generated by NP2+L could result in the release of DAMPs including ATP, CRT, and HMGB1, thereby inducing DC cell maturation, promoting the T‐cell infiltration, increasing the transformation of M2 macrophages to M1 macrophages, inhibiting the expression of Tregs cells in the tumor immune microenvironment, and increasing the PD‐L1 expression. Thus, the response rates of NP2+L combined PD‐L1 monoclonal antibodies improved.

Herein, a ROS‐responsive degradable polyphotosensitizer, i.e., a polymer with a photosensitizer molecular backbone, was designed (**P1**, **Scheme** **1**). This polyphotosensitizer was then derivatized with a new Michael addition acceptor into a new polymer (**P2**, Scheme 1). Furthermore, P1 and P2 were subsequently adopted to encapsulate a platinum (IV) prodrug of cisplatin (**Pt1**, Scheme 1) into ROS‐responsive NP1 and NP2 with photodynamic therapeutic effects, respectively. Under 808 nm NIR light irradiation, the dissociation of NP2, the light‐controlled release of cisplatin, the ability to inhibit GSTs activity and reduce the conjugation of GSH and cisplatin, and the process of inhibiting GST activity and reducing the cisplatin deactivation were investigated in vitro. Moreover, NP2+L was found to be able to generate ROS, induce type II ICD in tumor cells, generate strong immune responses, and, more importantly, increase PD‐L1 expression on the cancer cells. These findings indicate a possible synergy between NP2+L and PD‐L1 monoclonal antibodies. Furthermore, a mouse model of prostate cancer based on RM‐1 cells was constructed to evaluate NP2 in mice. NP2 could reach the tumor site via the enhanced permeability and enhanced retention (EPR) effect. More importantly, NP2+L could improve the response rates of PD‐L1 monoclonal antibody therapy, presenting with the best efficacy among multimodal treatments of combined photodynamic therapy, chemotherapy, and immunotherapy.

## 2 Results and Discussion

### 2.1 Preparation and Characterization of Nanoparticles

First, a platinum (IV) prodrug of cisplatin with two axial long aliphatic chains was synthesized by chemical modification of the dihydroxycisplatin (CisPt^IV^‐OH) with an anhydride (**Pt1**, Figures S1 and S2, Supporting Information). Subsequently, a Bodipy monomer with a double hydroxyl group that can generate photodynamic effects under light irradiation in the NIR I region was designed. This Bodipy monomer was further adopted to polymerize with a ROS‐sensitive 2,2′‐(propane‐2,2‐diylbis(sulfanediyl) bis(ethan‐1‐ol) (DSB) monomer as well as 1,6‐hexanediol and 1,2,4,5‐cyclohexanetetracarboxylic acid dianhydride (CHTA), which was then end‐capped with mPEG_5k_, thereby resulting in a biodegradable ROS‐sensitive amphiphilic polyphotosensitizer with photodynamic effects (**P1**, Scheme 1). Subsequent conjugation of a Michael addition acceptor, i.e., *N*‐(3‐aminopropyl) methacrylamide with the help of EDS/NHS with P1 resulted in the final polymer designated as P2 (Scheme 1). P1 and P2 were characterized by ^1^H NMR to confirm the successful synthesis (Figures S3 and S4, Supporting Information). The molecular weight of P2 was found to be 25 054 Da with fourteen Michael addition acceptors per polymer chain. On the one hand, P2 can produce ROS under the NIR I light irradiation. ROS generated by P2 can induce the breakage of its thioketal bonds, resulting in rapid degradation of P2. On the other hand, P2 with the Michael addition acceptor of *N*‐(3‐aminopropyl) methacrylamide could react with intracellular glutathione sulfhydryl transferases (electron donor), resulting in the reduction of the GSH‐mediated deactivation of cisplatin.

**Figure 1:**
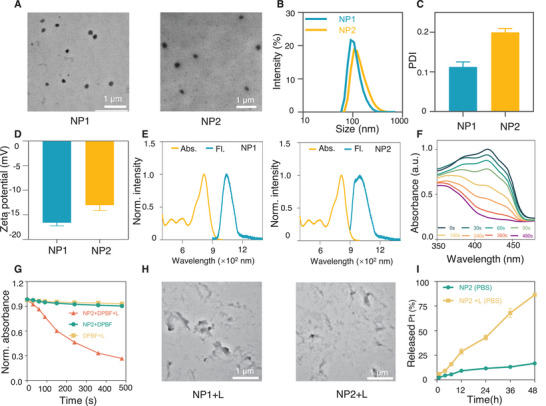
Preparation and characterization of NP1, NP2. A) TEM images of NP1 and NP2. B) The mean diameters, C) PDI and D) Zeta potentials of NP1 and NP2 by DLS. E) Absorption and fluorescence emission spectra of NP1 and NP2. F) Absorbance values of DPBF in the presence of NP2 under the 808 nm NIR light irradiation at different time points. G) Decomposition rates of DPBF by ROS generated from NP2 under NIR light irradiation (808 nm, 1.0 W cm^−2^, 2 min). H) Representative TEM images of NP1 and NP2 under the 808 nm NIR light irradiation. I) The cumulative Pt release in PBS from NP2 under 808 nm NIR light irradiation.

Further, P1 and P2 were adopted to encapsulate Pt1 respectively into nanoparticles, which could be designated as NP1 (containing Pt1 but not the Michael acceptor) and NP2 (containing Pt1 and Michael acceptor) via nanoprecipitation. Further, their physicochemical properties were investigated. First, by transmission electron microscope (TEM), NP1 and NP2 were found to have homogeneous spherical structures with average particle sizes at 146.5 and 184.0 nm, respectively (**Figure** **1**A). Meanwhile, by dynamic light scattering (DLS), the average particle sizes of NP1 and NP2 were found to be about 142.7 and 181.2 nm, respectively, and the PDIs were 0.112 and 0.200, respectively, indicating that both nanoparticles were well dispersed in aqueous solutions (Figure 1B,C). In addition, NP1 and NP2 had suitable zeta potentials at −16.60 and −13.03 mV, respectively (Figure 1D). In summary, relatively homogeneous nanoparticles were successfully prepared via nanoprecipitation.

Subsequently, the photophysical properties of NP1 and NP2 were then investigated using a UV–Vis spectrophotometer, and the results showed that the absorption peaks of both NP1 and NP2 were around 815 nm (Figure 1E), indicating that NP1 and NP2 could absorb light in the NIR I region. Meanwhile, both NP1 and NP2 were found to have NIR‐II fluorescence emission under 808 nm excitation, and their emission bands were mainly located in the range of 900–1400 nm (Figure 1E), implying that NP2 can be promising for NIR‐II bioimaging.

To further investigate the ability of NP2+L to generate ROS, the generated ROS was monitored with 1,3‐diphenylisobenzofuran (DPBF). Notably, DPBF has a strong absorption at 410 nm. However, in the presence of ^1^O_2_, DPBF undergoes a ring‐opening reaction, resulting in a decrease in its absorption at 410 nm. Therefore, the ability of photosensitizer to generate ^1^O_2_ under light irradiation can be reflected by how much the absorption peak of DPBF at 410 nm decreases.^[^ ^16^ ^]^ The results showed that the absorbance of the aqueous solution of DPBF in the presence of NP2 at 410 nm decreased rapidly with the longer light exposure time (Figure 1F; Figure S5, Supporting Information). Specifically, up to 73.3% of DPBF was oxidized after 480 s of light irradiation (Figure 1G). Taken together, the above results indicated that NP2+L could indeed generate ROS rapidly.

P2 contains thioketal bonds which can be broken by ROS.^[^ ^17^ ^]^ Therefore, ROS generated by NP2+L could result in nanoparticle dissociation and further trigger the release of the encapsulated Pt1. Therefore, the dissociation of NP2 as well as the platinum drug release under 808 lights were further investigated. On the one hand, we observed by TEM that NP2 underwent significant dissociation only after 2 min of light irradiation (808 nm, 1 W cm^−2^), and NP2 collapsed from the homogeneous spherical structure into an irregular morphology (Figure 1H). On the other hand, the release of platinum drug from NP2+L was monitored by an inductively coupled plasma‐mass spectrometry (ICP‐MS).^[^ ^18^ ^]^ The results showed that NP2 under NIR light irradiation (808 nm, 1 W cm^−2^,2 min) released a lot of platinum drugs in a time‐dependent manner. Specifically, 86.5% of the platinum was released within 48 h (Figure 1I). In summary, NP2+L could generate a large amount of ROS, and the generated ROS further induced the dissociation of NP2, resulting in the release of the encapsulated platinum drugs.

### 2.2 Cellular Uptake and ROS Generation by Nanoparticles

**Figure 2:**
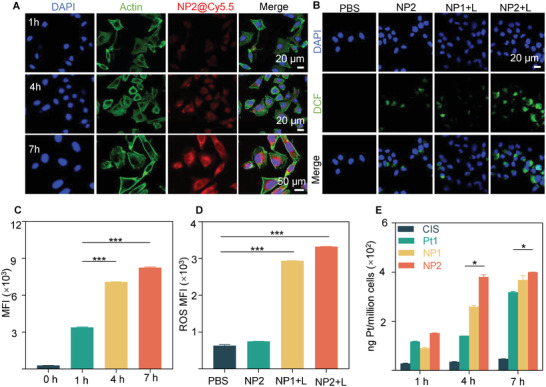
Endocytosis and ROS production by NPs in vitro. Endocytic uptake of NP2 detected by CLSM A) and flow cytometry C) in C4‐2 cells for 0, 1, 4, and 7 h. B) CLSM images of intracellular ROS level of C4‐2 cells. The green fluorescence was derived from DCF generated via the reaction of DCFH‐DA and ROS. Scale bar: 20 µm. D) Intracellular ROS generation with various treatments by FCM. E) Intracellular Pt uptake determined by ICP‐MS after treatment with various Pt‐containing drugs for 1, 4, and 7 h. Data are shown as mean ± SD. Statistical significance between the two groups was calculated via two‐way ANOVA and Tukey post hoc tests. *p < 0.05, **p < 0.01, ***p < 0.001.

Nanoparticles can exert their anticancer activity only after they are endocytosed by cancer cells.^[^ ^19^ ^]^ Therefore, to investigate the endocytosis of NP2 by prostate cancer cells, NP2 was first labeled with a Cy5.5 dye (NP2@Cy5.5). Next, the prostate cancer cells C4‐2 were observed by confocal laser microscopy (CLSM). The results showed that there was increasing red fluorescence in C4‐2 cells with the prolonged treatment time, indicating a time‐dependent intake of nanoparticles (**Figure** **2**A). Subsequently, the cellular intake of NP2@Cy5.5 was semiquantified by flow cytometry. The results confirmed the findings observed by CLSM. Specifically, a 1.4‐fold increase (*p* < 0.001) in uptake at 7 h was found in C4‐2 cells as compared to that at 1 h (Figure 2C; Figure S6, Supporting Information). In addition, it should be pointed out that there was a Pt element in NP2, making it possible to track the uptake of nanoparticles by cells quantitatively by ICP‐MS. The results showed that the platinum uptake in C4‐2 cells increased with the prolonged drug incubation time. Specifically, at 7 h, the intracellular Pt uptakes by C4‐2 cells treated with Cis, Pt1, NP1, and NP2 were 47.23 ± 0.31, 318.90 ± 1.88, 368.57 ± 11.98, and 400.15 ± 0.29 ng Pt/million cells, respectively, which was significantly higher than that of cells treated with Cis, Pt1, NP1, and NP2 at 1 h (28.90 ± 0.82, 117.72 ± 1.61, 90.92 ± 2.49, and 152.23 ± 0.60 ng Pt/million cells, respectively). In addition, the intracellular Pt uptake by cells treated with NP2 at 4 and 7 h was much higher than that by cells treated with Pt1 (*P* < 0.05) (Figure 2E), indicating NP2 was better taken up by C4‐2 cells.

The above study confirmed that NP2+L could efficiently generate ROS in vitro. To further investigate the ability of NP2+L to produce ROS in prostate cancer cells, on one hand, intracellular ROS production was directly observed by CLSM in cancer cells. DCFH‐DA is a reactive oxygen fluorescent probe, which can be oxidized by intracellular ROS to generate DCF, thus emitting strong green fluorescent products. The stronger the intracellular green fluorescence is, the more ROS is generated.^[^ ^20^ ^]^ Therefore, DCFH‐DA is frequently used as an intracellular probe for ROS. The results by CLSM showed that there was green fluorescence in cells treated with both NP1+L and NP2+L. However, it should be noted that there was greater and stronger green fluorescence in cells treated with NP2+L, indicating that more ROS was generated in this case. What's more, a semiquantitative analysis by FCM for the ROS generation in cells treated with NP1+L and NP2+L was performed. The results showed that the intracellular green fluorescence of cells treated with NP1+L and NP2+L was 4.6‐fold and 5.3‐fold higher than that of cells treated with PBS, respectively (Figure 1B,D; Figure S7, Supporting Information). The above results together indicated that both NP1 and NP2 could be rapidly taken up by cells and could generate ROS efficiently under NIR light irradiation.

### 2.3 Detection of Intracellular GSTs Activity

GSTs are involved in catalyzing the initiation step of the GSH conjugation reaction.^[^ ^3^ ^]^ A key reason why many tumors are resistant to cisplatin treatment is that cancer cells have overexpressed GST, which can rapidly bind to cisplatin entering the cells and inactivate the platinum drug. This process is also known as deactivation, which ultimately leads to the loss of cisplatin's anticancer activity.^[^ ^21^ ^]^ Therefore, inhibition of the activity of GSTs could inhibit the conjugation of GSH and platinum drugs, allowing more cisplatin to be retained to exert its effect.

**Figure 3:**
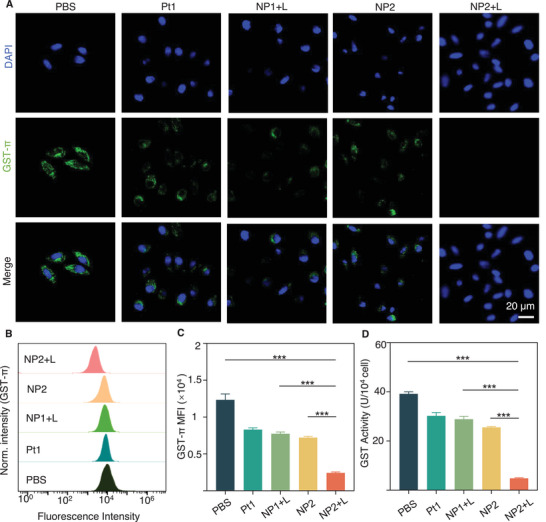
GST activity assays. A) CLSM images showing the expression of GST‐*π* (green fluorescence) in C4‐2 cells with various treatments. B) Flow curve for GST‐*π* expression in C4‐2 cells detected by flow cytometry. C) Quantitative analysis for GST‐*π* generation in C4‐2 cells detected by flow cytometry. D) GST‐*π* activity of C4‐2 cells with various treatments detected by GST ELISA kit after various treatments on C4‐2 cells. ***p < 0.001 determined by ordinary one‐way ANOVA and Tukey post hoc tests. Scale bar: 20 µm.

Here, a Michael addition acceptor of *N*‐(3‐aminopropyl) methacrylamide was conjugated to P2, which could bind to intracellular GSTs and inhibit the activity of GSTs, thereby inhibiting the cellular GSH conjugation. Therefore, the expression of GSTs was investigated in the cells receiving different drug treatments by CLSM. An indirect immunofluorescence staining to label the GSTs by the green fluorescent Alexa Fluor 488 secondary antibody was performed, and the intensity of the green fluorescence signal was used to indicate the GST's expression. The stronger the green fluorescence was, the more GSTs were in the cells. The results showed there was the weakest intracellular green fluorescence in C4‐2 cells treated with NP2+L, indicating there could be the lowest expression of GSTs (**Figure** **3**A). Subsequently, a semiquantitative analysis by flow cytometry was also performed: the expression of GSTs was reduced by 80.3% in C4‐2 cells treated with NP2+L compared to those treated with PBS (Figure 3B,C). Next, a GSTs activity assay kit was also used to detect the intracellular GSTs activity. It was found that the activity of intracellular GSTs in C4‐2 cells treated with NP2+L was reduced by 87.6% compared with that in cells treated with PBS (Figure 3D). The above results demonstrated that the Michael acceptor in NP2 can bind to intracellular GSTs and significantly reduce the GSTs activity in cancer cells, providing a great example of nanoparticles with a stronger antitumor activity.

### 2.4 In Vitro Antitumor Activity of NP2+L

**Figure 4:**
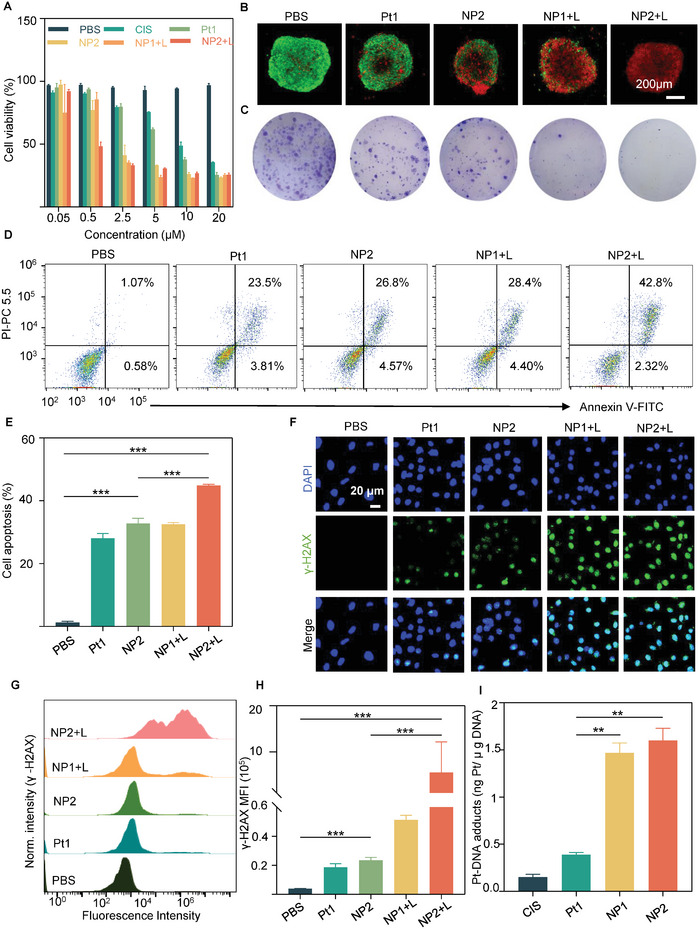
Antitumor activity and DNA damage of NP1 and NP2 in vitro. A) Cell viability of C4‐2 cells with various treatments by MTT. B) Representative CLSM images of C4‐2 tumor spheroids stained with Calcein‐AM (green, viable) and PI (red, dead) after being treated with different drugs. Scale bar: 200 µm. C) Colonies stained by crystal violet dye after different treatments. D) Flow cytometry scatter plot for apoptosis in C4‐2 cells detected by flow cytometry. E) Semiquantification of apoptotic rates of cells with different treatments at 24 h by flow cytometry. ***p < 0.001 determined by ordinary one‐way ANOVA and Tukey post hoc tests. F) Representative CLSM images of DNA damage in C4‐2 cells treated with different drugs (blue, DAPI; green, *γ*‐H2AX). Scale bar: 20 µm. G) Expression of *γ*‐H2AX in C4‐2 cells detected by flow cytometry. H) Semiquantification of *γ*‐H2AX expression in C4‐2 cells treated with different drugs for 24 h by FCM. ***p < 0.001 determined by ordinary one‐way ANOVA and Tukey post hoc tests. I) Quantification of Pt‐DNA adducts by ICP‐MS. **p < 0.01 determined by unpaired two‐sided *t*‐test. Data are shown as mean ± SD.

To investigate the antitumor activity of NP2+L, the cytotoxicity of prostate cancer cells treated with different drugs was first examined by an MTT assay. It was found that NP2+L was the most cytotoxic in all the prostate cancer cell lines, and the best antitumor effect was observed on C4‐2 cells, with a semi‐inhibitory concentration IC_50_ at 0.94 μM (**Figure** **4**A and Figure S8, Supporting Information). To visualize the antitumor effect of prostate cancer cells treated with different drugs, a 3D tumor spheroid model used for live and dead staining assay was constructed to visualize the anticancer activity of NP2+L. It was found that the number of dead cells (red) in the 3D tumor spheroid model treated with NP2+L was significantly more than that treated with other drugs, indicating that NP2+L had the best performance in killing cancer cells in the 3D tumor spheroid model (Figure 4B). Next, the anticancer effect of NP2+L was evaluated by a colony formation assay. The results showed that NP2+L significantly inhibited the colony formation of C4‐2 cells (Figure 4C). To further investigate the apoptosis in C4‐2 cells induced by NP2+L, the apoptosis of C4‐2 cells treated with different drugs was performed by an Annexin V‐FITC and propidium iodide (PI) double‐staining assay, and the apoptosis rate was statistically analyzed. The results showed that C4‐2 cells treated with NP2+L had the highest apoptosis rate of 44.90%, which was 1.37 times higher than that of C4‐2 cells treated with NP2, which was 32.78%, and also 1.38 times higher than that of C4‐2 cells treated with NP1+L at 32.53% (Figure 4D,E). The above results together suggested that NP2+L had better anticancer activity and could be efficient enough to promote apoptosis in prostate cancer cells.

It is well known that cisplatin can crosslink with DNA after entering the cancer cells, causing DNA damage, leading to cell cycle arrest and thus inducing cell death.^[^ ^22^ ^]^ To further investigate the damage to C4‐2 cells by NP2+L, the expression of *γ*‐H2AX (green fluorescence), a marker for DNA damage,^[^ ^23^ ^]^ was examined in the cells treated with NP2+L. The stronger the green fluorescence in the nucleus was, the more DNA damage there would be. First, an immunofluorescence staining assay (green) of *γ*‐H2AX in the nucleus of C4‐2 cells treated with different drugs was performed, and the CLSM results showed that the green fluorescence signal in the nucleus of C4‐2 cells treated with NP2+L was significantly higher than that in the nucleus of cells treated with other drugs, indicating that NP2+L resulted in the most profound DNA damage (Figure 4F). Subsequently, a semiquantitative analysis of *γ*‐H2AX expression in the nucleus C4‐2 cells treated with different drugs was performed by flow cytometry. The results revealed that C4‐2 cells treated with NP2+L resulted in 23.3 times more DNA damage than those treated with NP2 (Figure 4G,H).

It was shown that cisplatin‐induced apoptosis is mainly caused by the formation of Pt‐DNA adducts between cisplatin and DNA.^[^ ^24^ ^]^ Subsequently, the intracellular platinum‐DNA adducts in cells treated with different drugs were quantified by ICP‐MS. The results showed that there were more Pt‐DNA adducts in C4‐2 cells treated with NP2 and NP1 than those treated with Pt1 (Figure 4I). These results suggested that NP2 could crosslink with DNA in the cells, forming more platinum‐DNA adducts and causing more DNA damage.

### 2.5 An Immunogenic Cell Death Induced by NP2+L

**Figure 5:**
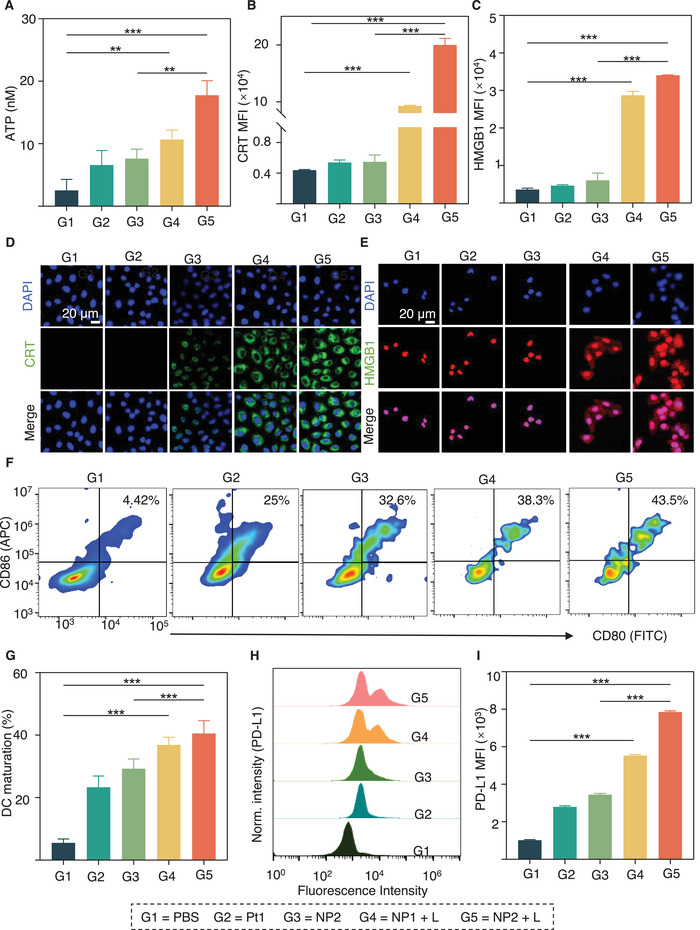
Confirmation that NP2+L contributes to immunogenic cell death in vitro. A) The quantitative examination of ATP secretion in the C4‐2 cells supernatants after various treatments. Quantification of CRT exposure B) and HMGB1 release C) by flow cytometry. CLSM images of CRT exposure D) and HMGB1 E) release on the surface of C4‐2 cells after various treatments. F) Flow cytometry scatter plot of DCs maturity in the RM‐1 cells detected by flow cytometry. G) Quantification of DCs maturity in the RM‐1 cells after various treatments. H) Representative flow cytometric curves and I) the corresponding quantification of PD‐L1 after different treatments. ***p < 0.001 determined by ordinary one‐way ANOVA and Tukey post hoc tests. Scale bar: 20 µm. Data are shown as mean ± SD.

The above studies confirmed that NP2+L could rapidly produce large amounts of ROS. A previous study showed that ROS could induce endoplasmic reticulum stress (ERS) in cells, thereby inducing type II ICD and the release of a series of damage‐associated molecular patterns (DAMPs).^[^ ^25^ ^]^ Among them, ATP released into the extracellular can promote the recruitment of antigen‐presenting cells (APCs) in tumor tissues; CRT transferred to the surface of tumor cells can contribute to the better recognition and uptake of tumor cells by dendritic cells (DCs); HMGB1 can be released from the nucleus to the cytoplasm to reduce the expression of tumor cell‐associated proteins, thus decreasing tumor proliferation, differentiation, and metastasis. Therefore, the content of ATP in C4‐2 cells cultures treated with NP2+L was first measured using an ATP assay kit. It was found that C4‐2 cells treated with NP2+L produced 7.0 times more ATP than those treated with PBS (**Figure 5A**) , indicating that NP2+L could significantly enhance extracellular ATP expression. Second, the outflows of CRT were quantified by flow cytometry (Figure 5B; Figure S9, Supporting Information). The results showed that the CRT exposure in cells treated with NP2+L (808 nm, 1 W cm^−2^, 2 min) was 36.45 times higher than that in cells treated with NP2, confirming that NP2+L could result in a stronger CRT exposure to C4‐2 cells. To better visualize the CRT exposure, C4‐2 cells treated with different drugs were also visualized by CLSM. The results showed that C4‐2 cells treated with NP2+L induced the strongest CRT exposure as there was the strongest green fluorescence (Figure 5D). Finally, the release of HMGB1 of C4‐2 cells was studied by CLSM via a semiqualitative analysis. As shown in Figure 5E, there was the strongest colocalization of DAPI (blue) and HMGB1 (red) fluorescence signals in C4‐2 cells treated with PBS. However, there was a large amount of HMGB1 expression observed in the cytoplasm of C4‐2 cells treated with NP2+L. Further, a semiquantitative analysis of HMGB1 release was also performed using flow cytometry. It was found that the expression of HMGB1 in the cytoplasm of C4‐2 cells treated with NP2+L was 5.62 times higher than that in cells treated with NP2 (Figure 5C; Figure S10, Supporting Information). Taken together, the above results indicated that NP2+L had a strong ability to induce HMGB1 release from the cell nucleus. To conclude, the above experimental results indicated that NP2+L was able to significantly induce the release of DAMPs for the subsequent activation of immune responses.

To further investigate the effect of NP2+L on the release of DMAPs and DC maturation, BMDCs were extracted from mice, which were then co‐incubated with pretreated C4‐2 cells according to the previous report.^[^ ^26^ ^]^ Subsequently, the DCs were examined and analyzed using flow cytometry. The results shown in Figure 5F,G demonstrated the highest percentage of DC maturation (CD80^+^CD86^+^) was up to 40.5% caused by coincubation of BMDC with NP2+L‐pretreated tumor cells, which was 1.38 times higher than that caused by coincubation with NP2‐ pretreated tumor cells. These results suggested that NP2+L could induce more DC maturation, providing a great possibility for subsequent activation of T cells’ recognition and attack on tumor cells and promotion of CD8^+^ T cell infiltration.

Programmed cell death‐ligand 1 (PD‐L1), a ligand protein produced by tumor cells, binds to PD‐1 in T lymphocytes, which transmits immunosuppressive signals, thereby reducing the activation and proliferation of CD8^+^ T cells in lymph nodes.^[^ ^27^ ^]^ Studies have shown that prostate cancer is generally an immune “cold tumor” with low PD‐L1 expression.^[^ ^28^ ^]^ Therefore, increasing the expression of PD‐L1 on the surface of prostate cancer cells may transform prostate cancer into an immune “hot tumor” and improve the response rate of immunotherapy.

Therefore, the effect of NP2+L on PD‐L1 expression was subsequently explored on the surface of C4‐2 cells. First, we found that the expression of PD‐L1 on the cell surface was significantly higher in C4‐2 cells treated with NP2+L by CLSM (red fluorescence indicating PD‐L1 expression) (Figure S11, Supporting Information). Subsequently, the expression of PD‐L1 on the surface of C4‐2 was also analyzed semiquantitatively by flow cytometry. The results revealed that the PD‐L1 expression of C4‐2 cells treated with NP2+L was 7.69 times higher than that of C4‐2 cells treated with PBS (Figure 5H,I). Taken together, NP2+L significantly increased the expression of PD‐L1 on the surface of prostate cancer cells, implying that NP2+L combined with PD‐L1 monoclonal antibody for prostate cancer could be promising in cancer therapy.

### 2.6 Biodistribution of NP2

**Figure 6:**
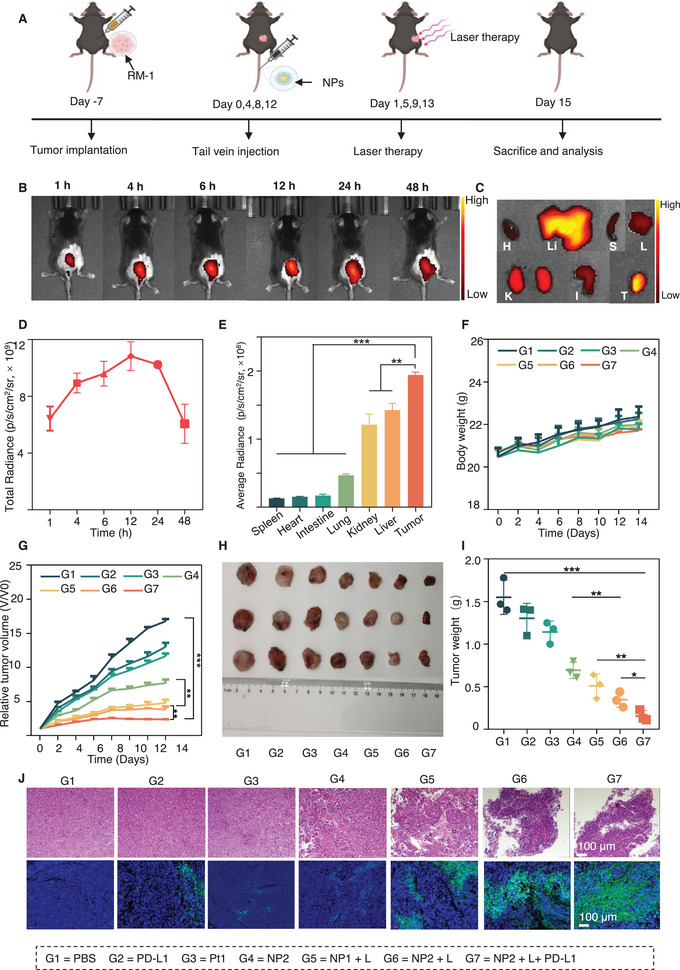
Biodistribution and anticancer effect of NP2 in mice bearing RM‐1 cancer. A) Schematic illustration of treatment schedule. B) Tumor imaging and D) quantification of fluorescence intensity of C4‐2 mice after intravenous injection of Cy5.5‐labeled NP2 at different times. C) Major organs imaging and E) quantification of fluorescence intensity of Cy5.5‐labeled NP2 48 h after intravenous injection. F) Body weight changes and G) tumor growth inhibition curves of mice in different groups. H) Representative tumor photograph images and corresponding tumor weight I) of different groups. J) H&E staining and TUNEL of tumor tissues. Scale bar: 100 µm. *p < 0.05, **p < 0.001, ***p < 0.001 determined by ordinary two‐way ANOVA and Tukey post hoc tests. *n* = 3 mice per group.

To investigate the biodistribution of NP2 in vivo, an RM‐1 subcutaneous tumor model was constructed (Figure 6A) . First, NP2 was labeled using Cy5.5 dye into NP2@Cy5.5 which was further used for the biodistribution study. The results showed that a distinct red fluorescence was detected at the tumor site by in vivo imaging system (IVIS) after the intravenous injection of NP2@Cy5.5 for 1 h. The fluorescence signal at the tumor site gradually enhanced over time (**Figure** **6**B). A semiquantitative study of the red fluorescence was then performed. It was found that the fluorescence signal at the tumor site reached its peak intensity after 12 h. At 24 h, the tumor site still had a high fluorescence signal (Figure 6D), indicating good targeting, accumulation, and retention of NP2@Cy5.5 at the prostate tumor sites. Subsequently, at 48 h, the mice were sacrificed and then observed by ex vivo imaging for the biodistribution of NP2@Cy5.5 in the major tissues and organs. The results showed that the fluorescence signal was strongest in tumor tissues and strong enough in major organs of metabolism, such as in liver and kidneys, ranking 2nd and 3rd, respectively, which were significantly higher than that in other organs such as the heart, lungs, spleen, and intestines (Figure 6C,E). The above results further suggested that NP2 was able to target and accumulate at the tumor sites, providing an important prerequisite for its in vivo antitumor applications.

### 2.7 Antitumor Effect of NP2 In Vivo

To further evaluate the antiprostate cancer effect of NP2+L in vivo, a mouse model of prostate cancer was established which was then treated according to the protocol as shown in Figure 6A. Moreover, the changes in tumor and body weights of mice were recorded on alternate days for two consecutive weeks. It was found that after 14 days of treatment with different drugs, the body weight of the mice treated with each drug changed, but the differences in body weight changes among mice were not significant (Figure 6F). In addition, no significant pathological changes or abnormalities in routine blood work were observed in the major tissues and organs of mice treated with each drug (Figures S12 and S13, Supporting Information). The above results implied that these drugs had no significant side effects. In terms of tumor suppression, the results showed that the tumor volume inhibition rate of NP2+L was 77.1% after 14 days of treatment compared with that of mice treated with PBS. Furthermore, the tumor inhibition rate further increased to 85.9% when NP2+L was used in combination with PD‐L1 monoclonal antibody (Figure 6G). In addition, the mice were sacrificed, and the tumors were removed and weighed. The results showed that the average tumor weight of mice treated with NP2+L was 0.35 g, which was 30.3% (1.55 g) of that of mice treated with Pt1. In particular, the average tumor weight of mice treated with NP2+L + PD‐L1 was only 13.7% (0.16 g) of that of mice treated with Pt1 (Figure 6H,I). These results indicated that tumor suppression by NP2+ L was significantly higher than that by Pt1 alone, and NP2+ L + PD‐L1 could synergistically produce a stronger tumor suppression.

Finally, the antitumor effects of NP2+L on mice were also verified by H&E and TUNEL staining, respectively. It was found that there was greater nuclear fragmentation and nucleolysis of tumor cells in mice treated with NP2 + L and NP2+ L + PD‐L1 than that in those treated with other drugs (Figure 6J, top panel). Further, TUNEL staining also indicated that the green fluorescence intensity (DNA damage area) in tumor tissue of mice treated with NP2+L and NP2+ L +PD‐L1 was stronger than that in mice treated with other drugs (Figure 6J, lower panel). In conclusion, NP2+L exhibited better antitumor ability in vivo, and the antitumor effect of NP2+L + PD‐L1 was even better.

### 2.8 NP2+L Induces an Immune Response In Vivo

To further investigate the ability of NP2+L to alter the tumor immune microenvironment and induce immune responses, the tumors, spleens, and lymph nodes of mice treated with different drugs were collected to analyze relevant immune parameters by CLSM and FCM, respectively.

**Figure 7:**
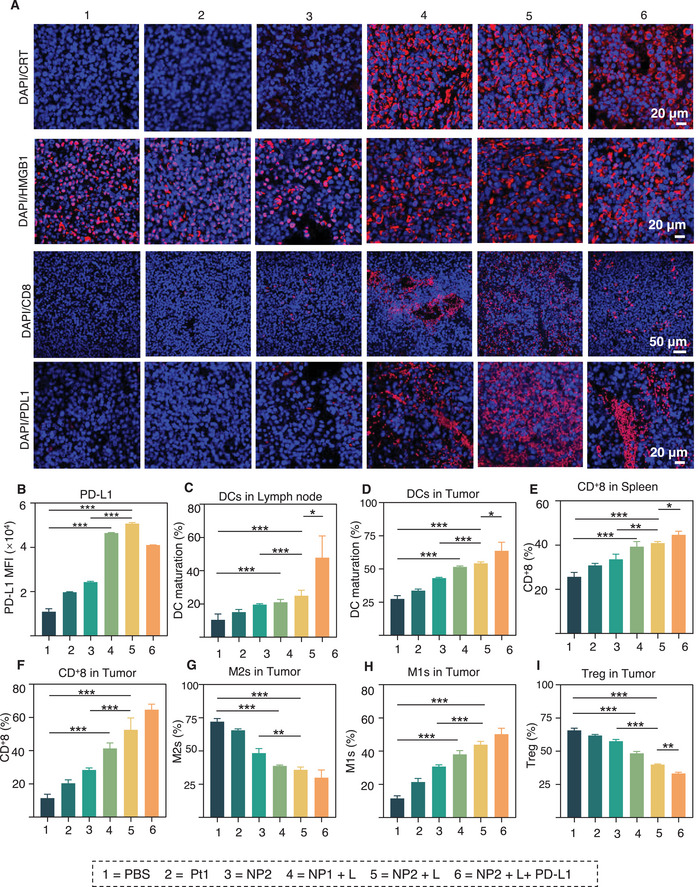
In vivo activation of systemic antitumor immune response triggered by NP2 + L. A) Immunofluorescence imaging of CRT, HMGB1, CD8^+^ T cell, and PD‐L1 in mice bearing RM‐1 tumor after different treatments. B) Quantification of PD‐L1 expression by flow cytometry in the RM‐1 tumor after various treatments. C,D) The percentages of mature DC populations (CD80^+^ CD86^+^) within RM‐1 tumors C) and lymph node. D) in each group are presented in histograms by flow cytometric analysis. E,F) The percentages of CD8^+^ T cell populations within RM‐1 tumors E) spleen F) in each group are presented as histograms. G,H) The proportions of M2 G) and M1 H) cells in each group are presented in histograms. I) The Treg populations in each group are presented in histograms. Data are presented as mean ± SD. Statistical significance between every two groups was calculated via one‐way ANOVA and Tukey post‐hoc tests. *n* = 3, *p < 0.05, **p < 0.01, ***p < 0.001.

The tumors were first collected for immunofluorescence staining. The results in **Figure** **7**A indicated that compared to tumor tissues from mice treated with other drugs, there was more CRT exposure (red fluorescence), HMGB1 migration (red fluorescence), infiltration of effector T cells (red fluorescence), and upregulation of PD‐L1 expression (red fluorescence) in tumors from mice treated with NP2+L, NP2+L + PD‐L1. The above results again demonstrated that NP2+L was able to induce more DMAPs release and migration, infiltration of immune cells, and upregulation of PD‐L1 expression.

Second, the immune‐related parameters of tumors, spleens, and lymph nodes in mice treated with different drugs were also analyzed using flow cytometry. It was found that the expression of PD‐L1 was increased in the tumor tissues of mice treated with NP2+L, which was 4.64 times higher than that in the mice treated with PBS. In addition, the expression of PD‐L1 in the tumor tissues of mice treated with NP2+L was 2.22 times higher than that in mice treated with NP2 (Figure 7B; Figure S14, Supporting Information). The above results in vivo are consistent with those found in the aforementioned in vitro study, which again validated the feasibility of NP2+L combined with PD‐L1 for cancer therapy.

The aforementioned study showed that NP2+L could significantly promote DC maturation in BMDC cells in vitro. The percentage of mature DCs (CD80^+^CD86^+^) in tumor tissues of mice treated with NP2+L in vivo was 25.0%, which was significantly higher than that in tumor tissues of PBS‐treated mice (10.5%) (*P* < 0.001) (Figure 7C; Figure S15, Supporting Information). More notably, the percentage of mature DC cells in tumor tissues of mice treated with NP2+L + PD‐L1 surprisingly reached 47.9%. This result also indicated that PD‐L1 could further stimulate the maturation of DCs, thus exerting a synergistic effect in NP2+L + PD‐L1.

Further, the percentage of mature DC cells in the lymph nodes of mice treated with NP2+L was 54.3%, which was 1.98 times higher than that of which in mice treated with PBS (Figure 7D; Figure S16, Supporting Information). The above results fully demonstrated that NP2+L could promote the maturation of DC cells in lymph nodes, providing the prerequisite for more efficient stimulation of T cell infiltration and increased anticancer activity of CD8^+^ T cells.

Next, effector T cell infiltration in the spleens and tumors of mice was investigated by FCM. The results showed that the proportions of CD8^+^T cells in the spleen of mice treated with NP2+L and NP2+L + PD‐L1 were 40.9% and 44.7%, respectively, which were 1.59 and 1.74 times higher than that in mice treated with PBS, respectively (Figure 7E; Figure S17, Supporting Information). In contrast, the proportions of CD8^+^ T cells in the tumor tissues of mice treated with NP2+L and NP2+L + PD‐L1 were 52.6% and 64.7%, respectively, which were 4.58 and 5.64 times higher than that in mice treated with PBS, respectively (Figure 7F; Figure S18, Supporting Information). The above results together indicated that NP2+L could significantly promote CD8^+^ T cell infiltration. Moreover, NP2+L + PD‐L1 further promoted the expression of CD8^+^T cells, thus exerting a stronger antitumor effect in vivo.

Tumor‐associated macrophages (TAM) are macrophages in tumor tissues, and activated macrophages mainly include M1‐type macrophages and M2‐type macrophages. M1‐type macrophages can promote inflammatory responses, kill intracellular pathogens and exert antitumor effects. In contrast, M2‐type macrophages can secrete inflammatory factors promoting immune escape and tumor progression.^[^ ^29^ ^]^ To investigate the effect of NP2+L on the distribution of M2‐type and M1‐type macrophages in tumor tissues, the ratio of two different phenotypes (M1: CD44^+^ CD62L^−^; M2: CD44^+^ CD62L^+^) in the TAMs of tumor tissues in mice treated with different drugs was examined using FCM. The results showed that the proportions of M1‐type macrophages and M2‐type macrophages in the tumor tissues of mice treated with NP2+L were 43.9% and 35.9%, respectively, with a 279.5% increase in M1‐type macrophages and a 50.2% decrease in M2‐type macrophages compared to that in mice treated with PBS. Finally, the distribution of two different phenotypes of cells in TAMs of tumor tissues in mice treated with NP2+L + PD‐L1 was further analyzed. The results showed that mice treated with NP2+L + PD‐L1 had a 334.0% increase in M1‐type macrophages and a 58.4% decrease in M2‐type macrophages, compared to mice treated with PBS. This also suggested that NP2+L + PD‐L1 caused a more pronounced trend of polarization of M2‐type macrophages to M1‐type macrophages in the TAMs of tumor tissues. The above results demonstrated that NP2+L could promote the polarization of M2 type macrophages to M1 type macrophages in TAMs, and NP2+L + PD‐L1 could do even better, with stronger antitumor effects (Figure 7G,H; Figure S19, Supporting Information).

Regulatory T cells (Tregs), as major immunosuppressive cells, can significantly inhibit the activation of antitumor immunity.^[^ ^30^ ^]^ To observe the effect of NP2+L on Tregs cells, the regulatory T cells (Tregs) in tumors of mice treated with different drugs were analyzed using flow cytometry. The results showed that the percentage of Treg cells in tumors of mice treated with NP2+L was significantly reduced, with a 39.1% decrease in Tregs compared with that in mice treated with PBS. This trend was even more pronounced in mice treated with NP2+L + PD‐L1 (49.4%) (Figure 7I; Figure S20, Supporting Information). The above results showed that NP2+L could inhibit the aggregation of Tregs in tumors, thereby inhibiting the tumor immune escape. Moreover, NP2+L + PD‐L1 could further inhibit tumor immune escape and exert stronger antitumor effects.

In summary, NP2+ L could induce a strong ICD, promote DC maturation and CD8^+^ T cell activation, encourage the polarization of M2 macrophages into M1 macrophages in TAMs, inhibit the aggregation of Treg cells in tumor tissues, and reshape the tumor immune microenvironment. Moreover, NP2+L + PD‐L1 can achieve better antitumor efficacy and enhance photodynamic immunotherapy.

## 3 Conclusions 

One of the main reasons for cisplatin resistance is the overexpression of GST in cancer cells, which promotes detoxification via GSH and cisplatin conjugation. In addition, very low response rates of many immune “cold tumors” limit the clinical application of immune checkpoint inhibitors. Herein, a ROS‐responsive biodegradable polyphotosensitizer, i.e., a polymer (P1) with photosensitizer molecular backbones, was designed. This polyphotosensitizer was further derivatized with a Michael addition acceptor into a new polymer (P2). P1 and P2 were adopted to encapsulate a platinum (IV) prodrug of cisplatin (Pt1), respectively, into ROS‐responsive nanoparticles NP1 and NP2 for photodynamic therapy (PDT).

In vitro, NP2 can be rapidly endocytosed and taken up by tumor cells, and a large amount of ROS can be produced rapidly under NIR light irradiation, which further leads to the dissociation and release of Pt1 from NP2. The Michael addition acceptor containing acrylamides in NP2 decreases GST activity by 87.6%, thereby greatly reducing the deactivation of cisplatin. This design in turn improves the ability of cisplatin to sustainably kill tumor cells and ultimately improves the effectiveness of chemotherapy. Therefore, NP2+L can efficiently kill prostate tumor cells via photodynamic therapy and chemotherapy with an IC_50_ value of only 0.94 μM. In vivo, NP2 could target tumors in mice. The tumor volume inhibition rate of NP2+L was 77.1% after 14 days of treatment. Furthermore, the tumor inhibition rate further increased to 85.9% when NP2+L was used in combination with PD‐L1 monoclonal antibody. In addition, NP2+L could induce type II ICD, further accelerating DC maturation, promoting CD8^+^ T cell infiltration, enabling M2 to M1 polarization of macrophages, inhibiting the expression of immunosuppressive Tregs, and increasing the expression of PD‐L1. NP2+L transformed the immune “cold tumor” into an immune “hot tumor” and further improved the anticancer efficacy of tumor immunotherapy in combination with PD‐L1monoclonal antibody.

Overall, the novel ROS‐responsive photodynamic nanoparticles with Michael addition acceptors delivering platinum (IV) prodrugs could efficiently target tumors in vivo and enhance the anticancer efficacy of chemotherapy and photodynamic immunotherapy for prostate tumors under NIR light irradiation, providing a new strategy for the clinical multimodal antitumor treatment in the near future.

### Ethics Approval Statement

This study was carried out in accordance with the relevant guidelines and regulations for the care and use of laboratory animals. All the animal procedures were approved by the Committee of Animal Experimentation and the Ethics Committee of Cancer Hospital, Chinese Academy of Medical Sciences (Approval number: NCC2022A142).

## Conflict of Interest

The authors declare no conflict of interest.

## Supporting information

Supporting Information

## Data Availability

The data that support the findings of this study are available from the corresponding author upon reasonable request.
