# Supplementary material for: Polyphotosensitizer‐Based Nanoparticles with Michael Addition Acceptors Inhibiting GST Activity and Cisplatin Deactivation for Enhanced Chemotherapy and Photodynamic Immunotherapy
Source: Adv Sci (Weinh). 2023 Mar 17;10(13):2300175. doi: 10.1002/advs.202300175 (PMC10161037; doi:10.1002/advs.202300175)
Supplement: Supplementary file 1 — Supporting Information [file ADVS-10-2300175-s001.pdf]

ADVANCED SCIENCE Open Access

Supporting Information

for *Adv. Sci.*, DOI 10.1002/advs.202300175

Polyphotosensitizer-Based Nanoparticles with Michael Addition Acceptors Inhibiting GST Activity and Cisplatin Deactivation for Enhanced Chemotherapy and Photodynamic Immunotherapy

*Qinxin Zhao, Ganghao Liang, Boda Guo, Wenkuan Wang, Chao Yang, Dong Chen, Feiya Yang *,Haihua Xiao* and Nianzeng Xing**

Supporting information

Polyphotosensitizers Based Nanoparticles with Michael Addition Acceptors Inhibiting GST Activity and Cisplatin Deactivation for Enhanced Chemotherapy and Photodynamic immunotherapy

Qinxin Zhao[1,2], *Ganghao Liang*[4,5], *Boda Guo*[1,2], *Wenkuan Wang*[1,2], *Chao Yang*[1,2], *Dong Chen*[1,2], *Feiya Yang*[1,2*], *Hauhau Xiao*[4,5*], *Nianzeng Xing*[1,2,3*]

[1] Department of Urology, National Cancer Center/National Clinical Research Center for Cancer/Cancer Hospital, Chinese Academy of Medical Sciences and Peking Union Medical College, 100021, Beijing, China.

[2] State Key Laboratory of Molecular Oncology, National Cancer Center/National Clinical Research Center for Cancer/Cancer Hospital, Chinese Academy of Medical Sciences and Peking Union Medical College, 100021, Beijing, China.

[3] Department of Urology, Shanxi Province Cancer Hospital/Shanxi Hospital Affiliated to Cancer Hospital, Chinese Academy of Medical Sciences/Cancer Hospital Affiliated to Shanxi Medical University, 030013, Shanxi, China.

[4] Beijing National Laboratory for Molecular Sciences, Laboratory of Polymer Physics and Chemistry, Institute of Chemistry, Chinese Academy of Sciences, Beijing 100190, China.

[5] University of Chinese Academy of Sciences, Beijing 100049, China.

TABLE OF CONTENTS

MATERIALS AND METHODS 3-14

SUPPLEMENTAL FIGURES

Figure S1 15
Figure S2 16
Figure S3 17
Figure S4 18
Figure S5 19
Figure S6 20
Figure S7 21
Figure S8 22
Figure S9 23
Figure S10 24
Figure S11 25
Figure S12 26
Figure S13 27
Figure S14 28
Figure S15 29
Figure S16 30
Figure S17 31
Figure S18 32
Figure S19 33
Figure S20 34

REFERENCES 35

Materials and Methods

Materials

Cisplatin (purity 99 %) was bought from Kunming Institute of Precious Metals, Yunnan, China. Hydrogen peroxide (H_2O_2) was purchased from Aladdin, Shanghai, China. n-Octanoic anhydride, 1,6-hexanediol, 1,2,4,5-cyclohexanetetracarboxylic acid dianhydride (CHTA), methoxy (polyethylene glycol)-5000 ($mPEG_{5000}$), anhydrous N, N-dimethylformamide, N-(3-aminopropyl) methacrylamide hydrochloride, 4-dimethylaminopyridine (DMAP), N-(3-dimethylaminopropyl)-N'-ethylcarbodiimide hydrochloride (EDCI), N-hydroxysuccinimide (NHS), triethylamine (TEA) was purchased from Energy Chemical, China. ^{1}H NMR spectra was measured by a 300 or 400 MHz NMR spectrometer (Bruker). The Size and zeta potential of nanoparticles were measured by a Malvern Zetasizer (Nano ZS, U.K.). High performance liquid chromatography (HPLC) analysis was performed on an Agilent 1200 series instrument. Transmission Electron Microscope (TEM, Hitachi HT7700, Japan) was used to observe the morphology of the nanoparticles.

Methods

Synthesis of CisPt^{IV}-OH

Cisplatin (300 mg, 1 mmol) was suspended in H_2O_2 (30 % w/v, 2.0 mL). The mixture was stirred at 40 °C overnight until the solution was clear. After cooling down to room temperature, a large amount of needle-like crystal was precipitated. The product was washed 3 times with cold ethanol and dried with a desiccator. The CisPt^{IV}-OH was isolated and yielded 80%.

Synthesis of Pt1

CisPt^{IV}-OH (200 mg, 0.6 mmol) was suspended in 10 mL anhydrous DMF. Then n-octanoic anhydride (649 mg, 2.4 mmol) was added. The reaction mixture was stirred overnight at 50 °C until the solution became clear. The solvent was removed under reduced pressure. Pt1 was recrystallized by MeOH to give a pale-yellow solid (yield, 53 %). ^{1}H NMR (400 MHz, DMSO-d_6): δ 6.52 (s, 6H), 2.20 (t, 4H, 7.5Hz),

1.44 (quin, 4H, 7.2Hz), 1.24 (m, 16H), 0.86 (t, 6H, 6.9 Hz).

Synthesis of P1

Bodipy monomer was prepared in a way our group reported recently[1]. 1,6-Hexanediol (0.156 mmol,18.4 mg), 2,2'-(propane-2,2-diylbis(sulfanediyl)) bis(ethan-1-ol) (DSB) (0.085 mmol, 16.6 mg), Bodipy monomer (0.042 mmol, 40 mg), and 1,2,4,5-cyclohexanetetracarboxylic acid dianhydride (CHTA) (0.298 mmol, 66.4 mg) were dissolved in 5 mL anhydrous DMF. After magnetic stirring at 50 ℃ for 24 h, $mPEG_{5000}$ (142 mg) was added to the reaction system and reacted for another 24 h. Subsequently, the mixture was placed in a dialysis bag (MWCO: 8000 Da) and dialyzed for 48 h. After 48 h, the solution was freeze-dried under reduced pressure to obtain P1 which was analyzed by ^{1}H NMR.

Synthesis of P2

P1 (ca. 0.25 mmol carboxyl group, 100 mg), DMAP (0.05 mmol, 6.1 mg), EDCI (0.5 mmol, 96 mg) and NHS (0.5 mmol, 58 mg) were dissolved in 4 mL DMF and stirred for 15 minutes. N-(3-aminopropyl) methacrylamide hydrochloride (0.275 mmol, 49 mg) was mixed with 52 μL TEA in a small EP tube, then the mixture was added to the above DMF solution and stirred overnight. Subsequently, the mixture was placed in a dialysis bag (MWCO: 8000 Da) and dialyzed for 48 hours. After 48h, the solution was freeze-dried under reduced pressure to obtain P2 which was analyzed by ^{1}H NMR.

Nanoparticles Formulation of NP1 and NP2

Briefly, a solution of Pt1 (10 mg) and P1 (18 mg) in DMSO (1 mL) was added to deionized water (18 mL) dropwise under vigorous stirring. The mixture was dialyzed in a dialysis bag (MWCO: 8000 Da) for 12 h. NP1 was obtained. NP2 was obtained in the same way as NP1.

Particle Characterization

^{1}H NMR spectra and ^{13}C NMR were measured by a 400 MHz NMR spectrometer (Bruker, Germany) at room temperature. The size distribution of NP was measured by dynamic light scatting analysis (DLS, Malvern Nano ZS90, UK). The morphology and element composition of NPs were characterized by transmission electron

microscope (TEM) (Hitachi HT 7700, Japan). The absorbance spectra and fluorescence spectra were measured using an ultraviolet-visible spectrometer (UV-vis, UV-2600, Shimadzu, Japan).

Detection of ROS generation in aqueous solution

Photo-triggered singlet oxygen generation (^{1}O2) of the NP2 was determined by using the chemical trapping method [2]. The 100 ug mL^{-1} NP2 were mixed with 1 ug mL^{-1} DPBF at a volume ratio of 1:40, followed by the analysis using a Microplate reader (SpectraMax, USA). The decomposition rate of DPBF by NP2 was recorded after different irradiation time durations (808 nm, 1.0 W cm^{-2}), the absorbance changes of DPBF at 415 nm was used to quantify decomposition rate.

Pt release of NP2

5 mL of NP2 under NIR irradiation for 2 minutes (808 nm, 1.0 W cm^{-2}) at a Pt concentration of 100 μM were transferred into a pre-swelled dialysis bag (MWCO: 3500 Da), which were then immersed into 200 mL PBS, 200 mL PBS in a shaking culture incubator at 37 °C respectively. At various time points, 1.5 mL of sample solution was withdrawn from the dialysate. Fresh corresponding solution (1.5 mL) was immediately added to the dialysate. All the samples were examined by ICP-MS. The platinum released from the micelles was expressed as the percentage of cumulative platinum in the dialysate to the total platinum in the nanoparticles.

Cell lines and culture

C4-2, PC3, and RM-1 cells, from American Type Culture Collection (ATCC), were cultured at 37 °C in RPMI 1640 medium (HyClone) with 10% fetal bovine serum (FBS) (Gibco) and 1% penicillin-streptomycin (Invitrogen) in a humidified incubator with 5% CO_2.

Intracellular ROS generation

Intracellular ROS level was investigated by using DCFH-DA as a fluorescent probe. In brief, a cover slide was placed in the bottom of each well of a 24-well plate. Cells (2×10^5) in 1 mL complete media were added to each well and incubated at 37 ℃ for 24 h. Afterward, the cells were treated with Pt1, NP2, NP1+L, NP2+L at the same

concentration (10 μM) for 12 h, respectively. The group of cells without any treatment were performed as negative control. Subsequently, the culture medium of the cells was replaced with a serum-free medium and then incubated with ROS indicator DCFH-DA (10 μM) for 30 mins. Then the cells of laser groups were irradiated with a NIR light irradiation for 2 minutes (808 nm, 1.0 W cm^{-2}) after washing with PBS. Afterward, the cover slide of each well was placed on the microslide, and the cell nuclei were stained with DAPI. Subsequently, images were collected with CLSM. Furthermore, the intracellular ROS level was further detected and quantified by Flow Cytometry (FCM). First, cells were seeded in 12-well plate at a density of 5×10^5 per well and incubated at 37 ℃ for 24 h. Afterward, the cells were treated with the same conditions as the above CLSM analysis. Finally, the cells were harvested to examine the intracellular DCFH-DA by FCM.

Nanoparticles uptake in the cells by confocal laser confocal scanning microscopy (CLSM) and flow cytometry (FCM)

A cover slide was placed in the bottom of each well of a 24-well plate. C4-2 cells (2×10^4) in 1 mL media were added to each well and incubated at 37 °C for 24 h. Then the cells were treated with Cy5.5-labeled NP2@Cy5.5 at a final concentration of 2 μg/ml for 0h, 1 h, 4 h or 7 h respectively. After being washed with cold PBS, the cells were fixed with paraformaldehyde. Cell nuclei were stained with DAPI. Subsequently, images were observed with CLSM. To perform flow cytometry, C4-2 cells were seeded in six-well plates at a density of 5×10^5 per well and incubated at 37 °C for 24 h. Then the cells were treated with Cy5.5-labeled NP2@Cy5.5 at a final concentration of 2 μg/ml for 0h, 1 h, 4 h or 7 h respectively. Finally, the cells were harvested to examine the intracellular uptake by FCM.

Study of the Uptake of Various Pt Containing Drugs in C4-2 Cells

C4-2 cells(1×10^6) were seeded in 6-well plates and cultured for 24 h. Then, different Pt containing drugs including cisplatin, Pt1 (Pt (IV)), NP1 and NP2 were added to the wells at the same concentration (10 μM) for 0 h, 1 h, 4 h or 7 h, respectively. The cells were then washed with PBS three times and then treated with nitric acid (100 ul),

H_2O_2 (100 ul) and aqueous solution containing 1% nitric acid (1.8 ml) for 24 h. Thereafter, Pt content of each sample was measured by ICP-MS.

Determine the anticancer activity of Pt (IV) drugs with cell *via*bility assays

Three prostate cancer cell lines (C4-2, PC3 and RM-1) were seeded in 96-well plates (5×10^3 cells/well) and incubated with RPMI1640 supplemented with 10% FBS and 1% penicillin-streptomycin at 37 °C for 24h. Then the cells were treated with PBS, cisplatin, Pt1, NP2, NP1L and NP2L at various concentrations of Pt ranging from 0.05 µM to 20 µM. Thereafter, the cellular viability was assessed *via* an MTT (3-(4,5-Dimethylthiazol-2-yl)-2,5-Diphenyltetrazolium Bromide) colorimetric assay. The MTT assay was performed 24 h after drug exposure. Then, MTT reagent (10 µL of a 5 mg/mL solution in PBS buffer) was added to each well and the plates were incubated for another 4 h. Acidified SDS solution was then added (100 µL/well). The plates were kept in the dark for an additional 12 h. Measurements of absorbance were subsequently completed with a Bio-Rad plate reader (SpetraMax M3) at 570 nm (peak absorbance) and at 650 nm (background absorbance).

Live/Dead Cell Staining

C4-2 cells were treated with the different drug treatment and laser irradiation (808 nm, 1.0 W cm^{-2}). The treated cells were then stained with a PBS buffer containing Calcein-AM and PI for 30 min in the cell-cultured container. After PBS wash, the cell samples were imaged on CLSM with the excitation at 488 nm and 532 nm, respectively.

Colony formation assays

C4-2 cells were seeded in 6-well plates at a density of 2×10^3 cells per well and cultured for 24 h. Subsequently, the cells were treated with PBS, Pt1, NP2, NP1L and NP2L (Pt1 µM). Then, the medium was refreshed for 7 days incubation before s taining with 0.2% crystal violet (Beyotime).

Apoptosis analysis

Cellular apoptosis was assessed with an Annexin V-FITC apoptosis detection kit (Beyotime) according to the manufacturer's instructions. In brief, C4-2 cells were

seeded on 12-well plates at 5×10^5 cells per well. After 24 h incubation, cells were treated with PBS, Pt1, NP2, NP1L and NP2L (all at 10 μM) for 24 h, washed with PBS, and incubated with Annexin/PI reagent in the dark for 15 min at 25 °C. Thereafter, the cells were immediately measured with FCM.

Nanoparticles induced DNA damage in the cells detected by confocal laser confocal scanning microscopy (CLSM) and flow cytometry (FCM)

A cover slide was placed in the bottom of each well of a 24-well plate. C4-2 cells (2×10^4) in1 mL media were added to each well and incubated at 37 °C for 24 h. Then the cells were treated with PBS, Pt1, NP2, NP1L and NP2L for 24 h, respectively. Then the cells were washed with PBS, fixed with paraformaldehyde, blocked with 1% BSA (Beyotime), and penetrated by 0.1% Triton (Beyotime). Then incubated with γ-H2AX overnight at 4 °C. The next day, the C4-2 cells were washed three times with PBS and then incubated with Alexa Fluor 488-conjugated antibody (ab150077, Abcam) for 2 h at room temperature. After being washed with cold PBS again, cell nuclei were stained with DAPI for 10 minutes at room temperature. Subsequently, images were collected with CLSM. To perform flow cytometry, C4-2 cells were seeded in six-well plates at a density of 5×10^5 per well and incubated at 37 °C for 24 h. The next processing steps are the same as the CLSM method. Finally, the cells were harvested to examine the expression of γ-H2AX by FCM.

Detection of Pt-DNA adducts in cells

C4-2 cells were seeded into 6-well plates at a density of 1×10^6 cells per well and incubated at 37 °C for 24 h. CisPt, Pt1, NP1 and NP2 were added to the wells at a concentration of 10 μM. After incubation for 12 h, the plates were replaced with fresh medium and cultured for another 12 h. Subsequently, the cells were collected and centrifuged, followed by washing with cold PBS three times. Then, DNA was extracted using a Cells Genomic DNA Extraction Kit (Solarbio, D1700). DNA concentration was measured by a NanoDrop 2000 spectrophotometer (Thermo Fisher Scientific, USA), and Pt content was determined *via* ICP-MS. The DNA-Pt adducts were quantitatively expressed as ng Pt per mg DNA.

Detection of GST-π

A cover slide was placed in the bottom of each well of a 24-well plate. C4-2 cells (2×10^4) in1 mL media were added to each well and incubated at 37 °C for 24 h. Then the cells were treated with PBS, Pt1, NP2, NP1L and NP2L for 24 h, respectively. Then the cells were washed with PBS, fixed with paraformaldehyde, blocked with 1% BSA (Beyotime) for 1 h. Then incubated with GST-π (ab138491, Abcam) overnight at 4 °C. After C4-2 cells were washed three times with PBS, and then incubated with Alexa Fluor 488-conjugated antibody (ab150077, Abcam) for 2 h at room temperature. After being washed with cold PBS again, cell nuclei were stained with DAPI for 10 minutes at room temperature. Subsequently, images were collected with CLSM. To perform flow cytometry, C4-2 cells were seeded in six-well plates at a density of 5×105 per well and incubated at 37 °C for 24 h. The next processing steps are the same as the CLSM method. Finally, the cells were harvested to examine the expression of GST-π by FCM.

Detection of glutathione S-transferase (GSTs) activity

C4-2 cells (1×10^6) were seeded into 6-well plates and further cultured for 24 h. After that, the cells were treated with PBS, Pt1, NP2, NP1L and NP2L for 24 h, respectively. After 12 h, the ells were collected into a 1.5-ml centrifuge tube and detected the activity of GSTs according to the operating instructions of the glutathione s-transferase kit. To put it simply, reagent 1 (10^5 cells/ml) was added in different groups, 3min was crushed by ultrasonic in ice bath, then centrifuged at 4 ℃ for 8000 g for 10 min, and the supernatant was placed on ice to be tested. Add the reagents specified in the instructions in turn to the trace quartz colorimetric plate / 96-well UV plate. Then the absorbance at 340nm was measured in 10 seconds and 310 seconds. Then calculate the activity of GST according to the formula in the manual. All experiments were carried out in triplicate.

Immunogenic cell death *in vitro*

To evaluate immunogenic cell death (ICD) induced by different drug treatments in prostate cancer cells, secretion of adenosine triphosphate (ATP), calreticulin (CRT)

exposure, and extracellular release of high mobility group box 1 (HMGB1) were examined *in vitro*. Extracellular secretion of ATP was evaluated with a commercially available ATP assay kit (S0026, Beyotime). Briefly, cells were seeded in 12-well plate at a density of 2 x10^5 cells/well. After incubated for 12 h, the cells were treated with PBS, Pt1, NP2, NP1L and NP2L for 24 h, respectively. Afterward, the culture medium was collected and the concentration of ATP was evaluated with an ATP assay kit according to manufacturer's instructions.CRT exposure was evaluated by FCM and CLSM. For FCM analysis, cells were seeded in the12-well plate at a density of 2 x10^5 cells/well. After 12 h pre-incubation, the cells were treated with PBS, Pt1, NP2, NP1L and NP2L for 6 h, respectively. Then the cells were washed with PBS and further incubated with CRT antibody (ab92516, Abcam). After that, the cells were incubated with Goat Anti-Rabbit IgG H&L (Alexa Fluor® 488) (ab150077, Abcam), washed with PBS, collected and suspended in PBS. Finally, the surface fluorescence was assayed with FCM. For CLSM analysis, a cover slide was placed in the bottom of each well of a 24-well plate. Cells (2 x10^4) in 1 mL complete media were added to each well and incubated at 37 ℃ for 12 h. Then the cells were treated with PBS, Pt1, NP2, NP1L and NP2L for 6 h, respectively. Next, the cells were fixed with 4% paraformaldehyde for 10 min, followed by incubation with 1% BSA in PBS for 1h. Then the cells were incubated with primary CRT antibody for night at 4 °C, and then incubated with the Alexa Fluor® 488-conjugated secondary antibody after three washes with PBS. Finally, the cells were stained with DAPI, and observed under CLSM using 405 nm and 488 nm lasers for visualizing nuclei and CRT exposure on the cell membrane, respectively. For evaluating intracellular HMGB1, a cover slide was placed in the bottom of each well of 24-well plate. Cells (2 x10^4) in 1 mL complete media were added to each well. After 12 h pre-incubation, the cells were treated with different formulations like the CRT test for 24 h. After that, the cells were washed with PBS for three times. Then, the cells were fixed with 4% paraformaldehyde for 10 min and permeabilized with 0.1% Triton X-100 for 10 min, followed by incubation with 1% BSA in PBS for 1h. Next, the cells were incubated

with primary HMGB1 antibody (ab79823, Abcam) for night at 4 °C, and then incubated with the 555-conjugated secondary antibody (ab150078, Abcam) after three washes with PBS. Finally, the cells were stained with DAPI and examined by CLSM. The expression of HMGB1 release assessed by flow cytometry was essentially the same as the method described above.

Dendritic cells activation *in vitro*

To evaluate dendritic cells (DCs) activation *in vitro*, bone marrow-derived dendritic cells (BMDCs) were obtained from the bone marrow of mice. RM-1 cells were first treated with PBS, Pt1, NP2, NP1L and NP2L for 24 h. Afterwards, BMDCs were cultured with 1.5 mL fresh medium and 1.5 mL tumor cells cultured medium obtained from different pretreated RM-1 cells groups. After that, the BMDCs were collected and stained with anti-CD11c, anti-CD80 and anti-CD86 antibodies. Finally, the activation of DCs was examined by FCM.

Detection of PDL-1 expression *in vitro*

A cover slide was placed in the bottom of each well of a 24-well plate. C4-2 cells (2×10^4) in1 mL media were added to each well and incubated at 37 °C for 24 h. Then the cells were treated with PBS, Pt1, NP2, NP1L and NP2L for 24 h, respectively. Then the cells were washed with PBS, fixed with paraformaldehyde, blocked with 1% BSA (Beyotime) for 1 h. Then incubated with PD-L1 (ab213524, Abcam) overnight at 4 °C. After C4-2 cells were washed three times with PBS, and then incubated with Alexa Fluor 488-conjugated antibody (ab150077, Abcam) for 2 h at room temperature. After being washed with cold PBS again, cell nuclei were stained with DAPI for 10 minutes at room temperature. Subsequently, images were collected with CLSM. To perform flow cytometry, C4-2 cells were seeded in six-well plates at a density of 5×105 per well and incubated at 37 °C for 24 h. The next processing steps are the same as the CLSM method. Finally, the cells were harvested to examine the expression of PD-L1 by FCM.

Animal welfare and protocols

Healthy male C57BL/6 mice were purchased from Beijing Vital River Laboratory Animal Technology Co., Ltd. (Beijing, China) and raised in specific pathogen-free (SPF) animal rooms. This study was carried out in accordance with the relevant guidelines and regulations for the care and use of laboratory animals. All the animal procedures were approved by the Committee of Animal Experimentation and the Ethics Committee of Cancer Hospital, Chinese Academy of Medical Sciences (Approval number: NCC2022A142).

Tumor model and biodistribution of NP2 *in vivo*

Male C57BL/6 mice were subcutaneously inoculated with 100 μL of RM-1 cells (2×10^6 cells) on the right hind leg. RM-1 tumor-bearing mice were intravenously injected with Cy5.5 labeled NP2 (2mg/kg) when the tumor volume reached about 200 mm^3, followed by imaging with an IVIS Spectrum (PerkinElmer) at 1 h, 2 h, 4 h, 6 h, 12 h, 24 h, 48 h and 72 h post injection, respectively (excitation wavelength: 740 nm, fluorescence emission signal wavelength: 800 nm). Mice were sacrificed to collect tumors and major organs for *ex vivo* imaging 48 h post injection.

Therapeutic effect of NPs on RM-1 models

When the subcutaneous RM-1 tumors reached approximately 100 mm^3, the mice were randomly divided into seven groups with 3 mice in each group. The drugs were i.v. injected once every 3 days for 4 times. Treatment groups were: 1) Control (200 μL), 2) PDL-1 (10 mg/kg), 3) various Pt containing drugs (3 mg Pt/kg). The body weight and tumor volume were recorded every other day. The tumor volume was calculated following the equation below: Tumor volume = $1/2 \times LW^2$, "L" referred to the long diameter of the tumor. "W" meant the short diameter of the tumor. The relative tumor volume was set as the V_t/V_o. Vt referred to the mean tumor volume at a desirable day when tumor volumes were collected. Vo referred to total mean value of the initial

tumor volume.

Histopathological analysis

The solid tumor and major organs were harvested from tumor-bearing mice on the 15th day of tumor inoculation for histological observation by standard H&E staining and immunofluorescence staining. For H&E staining, the excised tumor and organs were fixed in 4% paraformaldehyde solution, embedded in paraffin, sectioned, and stained with hematoxylin and eosin (H&E). The sections were then observed under a fluorescence microscope (IX83, Olympus). TUNEL staining was also used to evaluate apoptosis of tumor tissues, the procedures were consistent with the manufacturer's protocol, and finally examined by CLSM. For detecting the expression of CRT exposure, the release of HMGB1, infiltration of $CD8^+$ T cells and the expression of PD-L1 in tumor tissues, frozen tumor sections were fixed, permeabilized (HMGB1), and blocked with 3% BSA. Then the sections were incubated with primary antibodies against CRT (ab92516, Abcam), HMGB1(ab79823, Abcam), CD8 (MA1-84018, Invitrogen) and PD-L1 (ab213524, Abcam) overnight at 4 °C, followed by processing with second antibodies. Nuclei were counterstained with DAPI (ThermoFishere Scientific) and then the stained sections were imaged with a confocal microscope (LSM880, Zeiss). Cells were processed in the same way as described above for immunofluorescence.

Flow cytometry analysis of the animal tissue

The obtained fresh tumors, tumor-draining lymph nodes and spleens were collected for antitumor immune response analysis *via* FCM. The cells were stained by anti-CD274 (B7-H1, PD-L1)-PE, anti-CD45-FITC for FCM analysis. To detect the impacts on DCs maturation in tumor and lymph nodes, the tumor tissues from each treatment were collected and homogenized into single cell suspension. The cells were stained by anti-CD11c-PE, anti-CD80-FITC and anti-CD86-APC for FCM analysis. To detect the infiltration of antitumor T cells, the isolated cell suspensions were

incubated with anti-PC5.5-CD3 or anti-PE-CD3, anti-CD8-FITC and anti-CD4-APC for FCM analysis. The $CD8^+$ T cells were marked as $CD3^+CD4^-CD8^+$ T cells, which was presented as the percentage of $CD4^-CD8^+$ cells in $CD3^+$ T cells. To detect the ability on eliminating M2 type TAMs and increasing M1 macrophages, the cell suspensions were incubated with anti-F4/80-PE, anti-CD80-FITC and anti-CD206-APC according to the manufacturer's protocols. The cell population of M2 phenotype TAMs (characterized as $CD80^-$ $CD206^+$ cells) and M1 phenotype macrophages (characterized as $CD80^+CD206^-$ cells) were determined using FCM analysis. Finally, Tregs (CD4+$Foxp3^+$) in tumor tissues were also evaluated.

Statistical analysis

GraphPad prism 8.0.1 (GraphPad Softwareia) was used for all statistical analyses. The raw data was pre-processed by transformation or normalization. Data were presented as mean ± SD from at least 3 independent experiments of biological replicates, if not stated in the figure legend. Unpaired two-sided t-test was used between two groups, one-way or two-way analysis of variance (ANOVA)and Tukey post-hoc tests were used when more than two or multiple groups were compared. The type of test, number of independent repeats, as well as level of significance is mentioned in the caption of each figure/experiment. The level of significance was set at $P < 0.05$. * $p < 0.05$, ** $p < 0.01$, *** $p < 0.001$.

SUPPLEMENTAL FIGURES

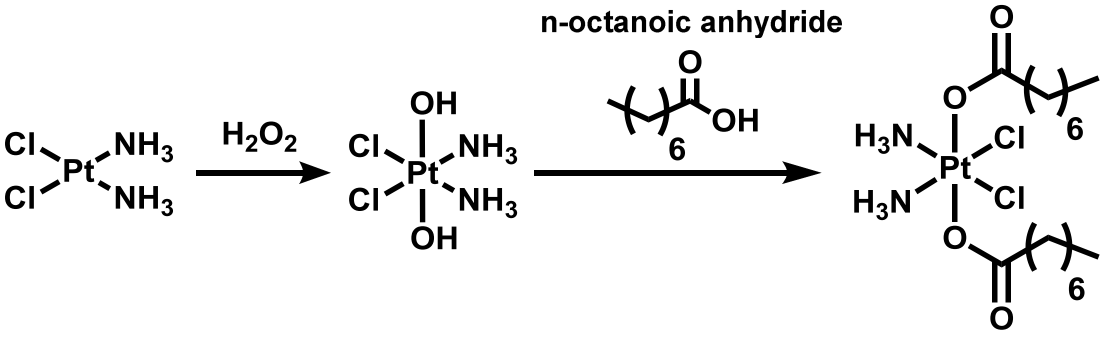

Figure S1. Synthetic route of Pt1.

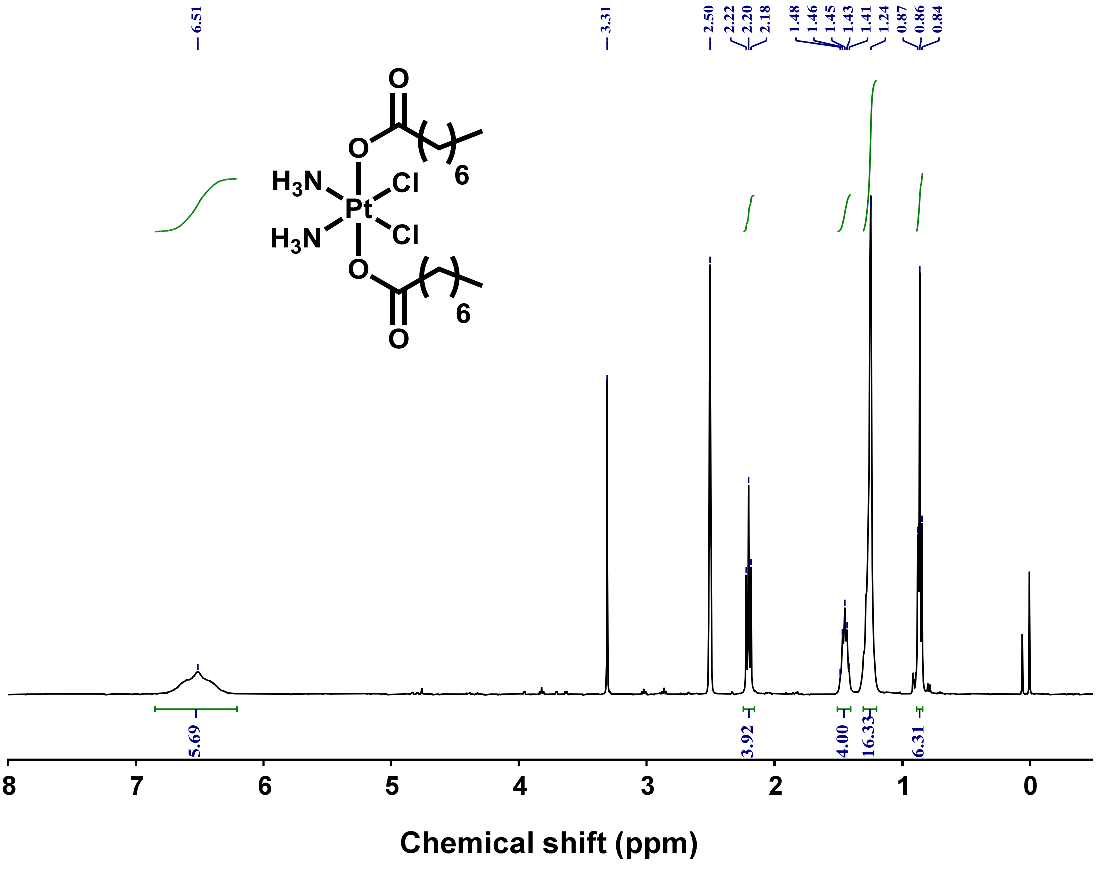

Figure S2. ^{1}H NMR spectrum of Pt1 in DMSO-d_6.

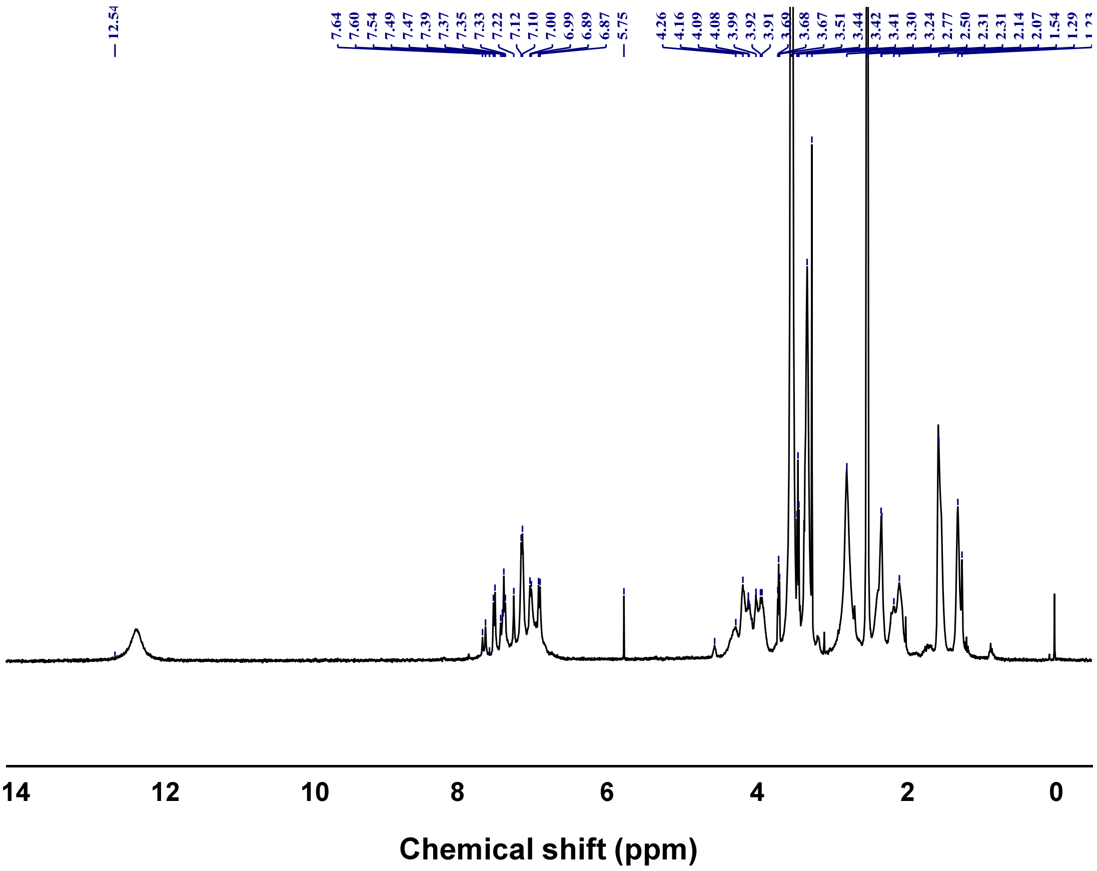

Figure S3. Characterization of P1 by ^{1}H NMR

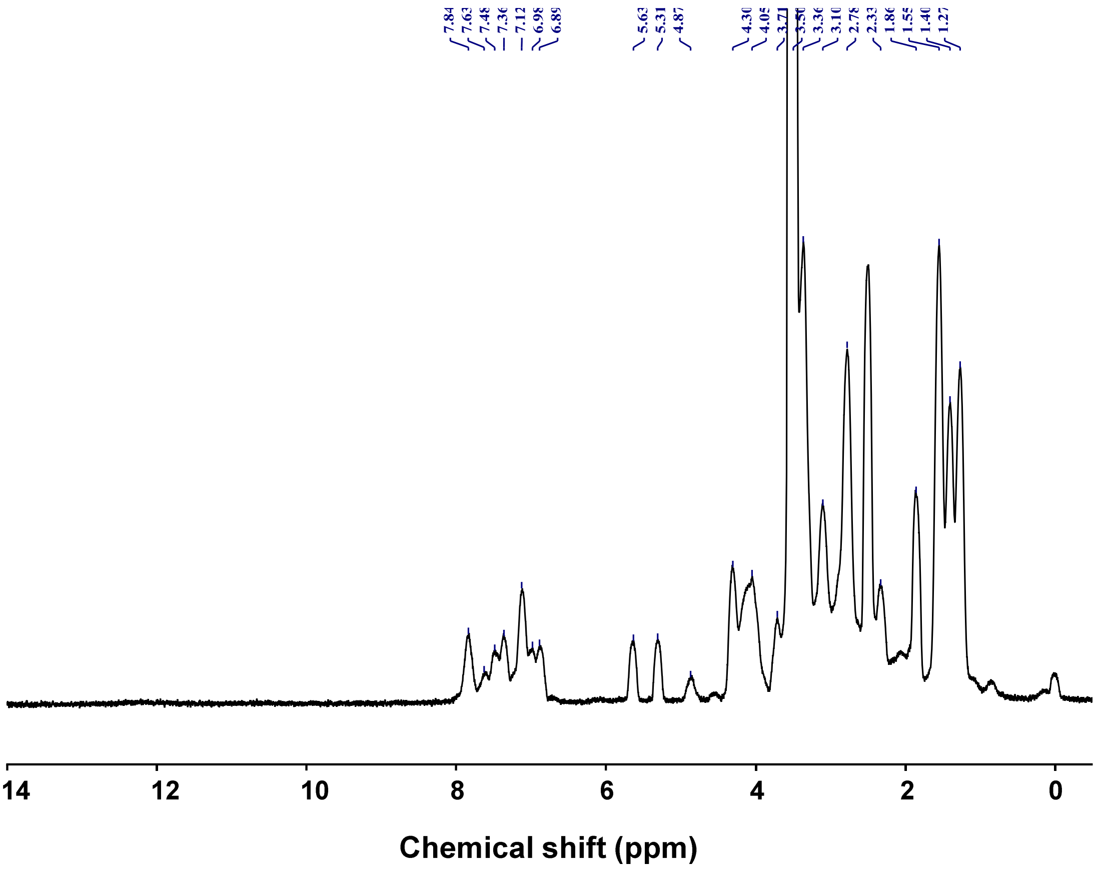

Figure S4. Characterization of P2 by ^{1}H NMR

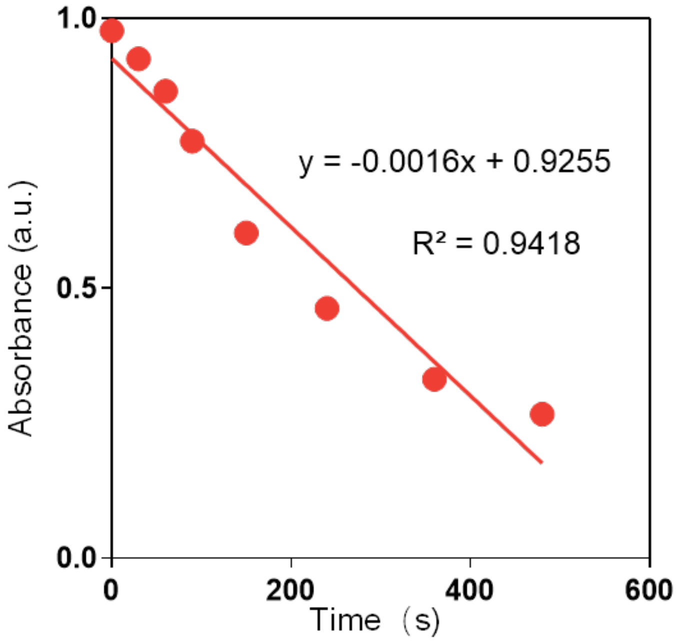

Figure S5. Absorbance values of DPBF by ROS under NIR light irradiation at different times.

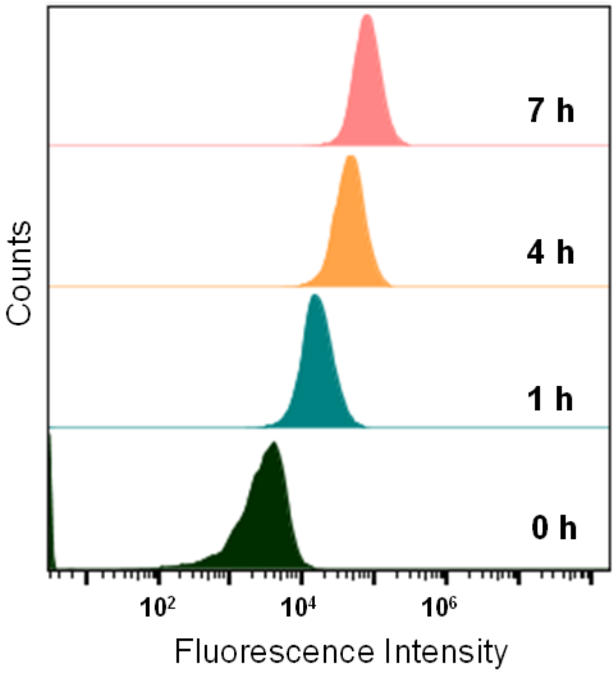

Figure S6. Endocytic uptake of NP2 detected by flow cytometry in C4-2 cells for 0 h, 1 h, 4 h, and 7 h.

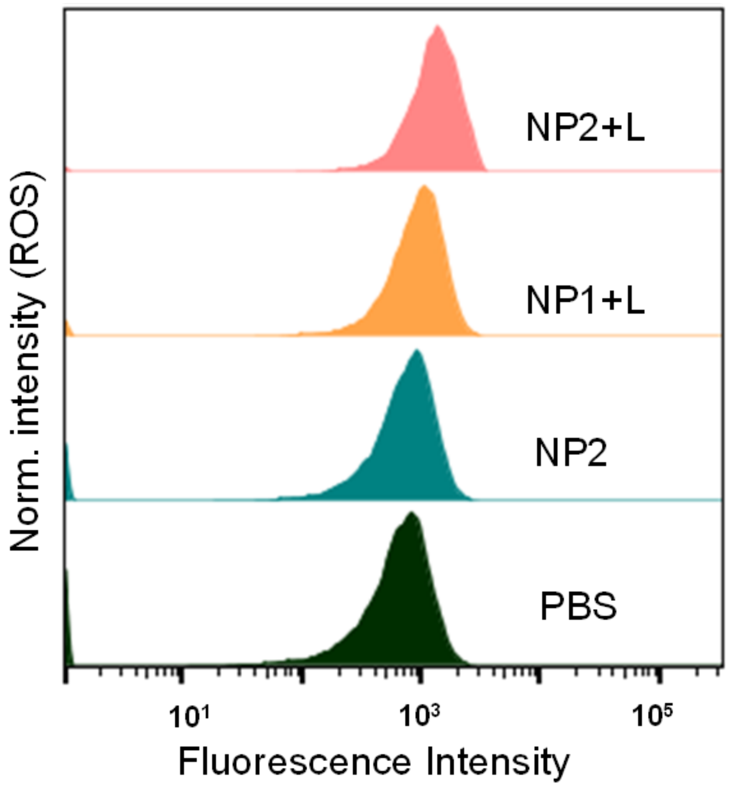

Figure S7. Flow curve for ROS expression in C4-2 cells detected by flow cytometry.

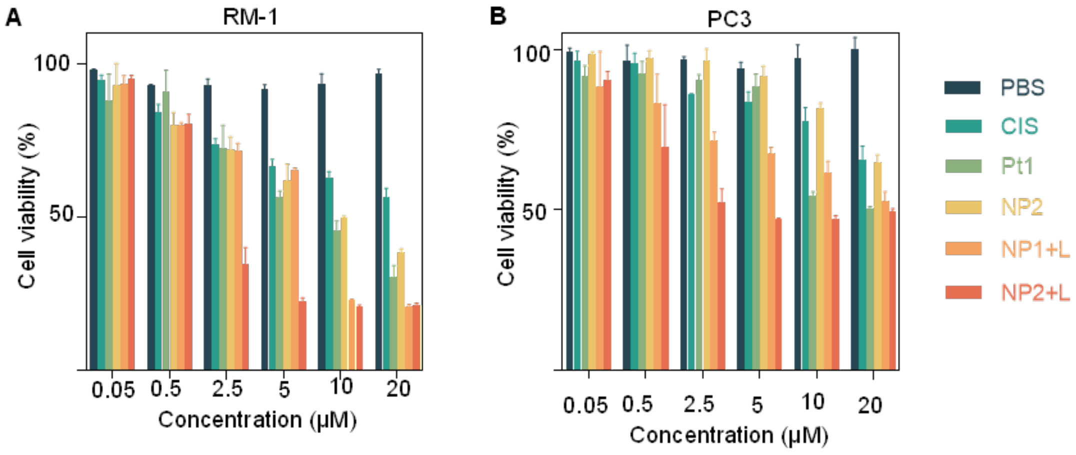

Figure S8. Cell viability of RM-1 and PC3 cells with various treatments by MTT

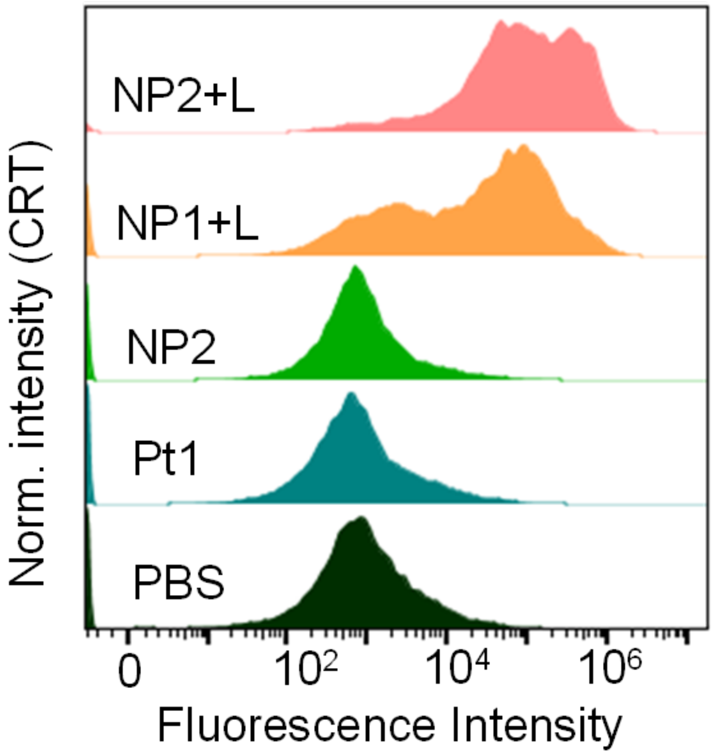

Figure S9. Flow curve for CRT expression in C4-2 cells detected by flow cytometry.

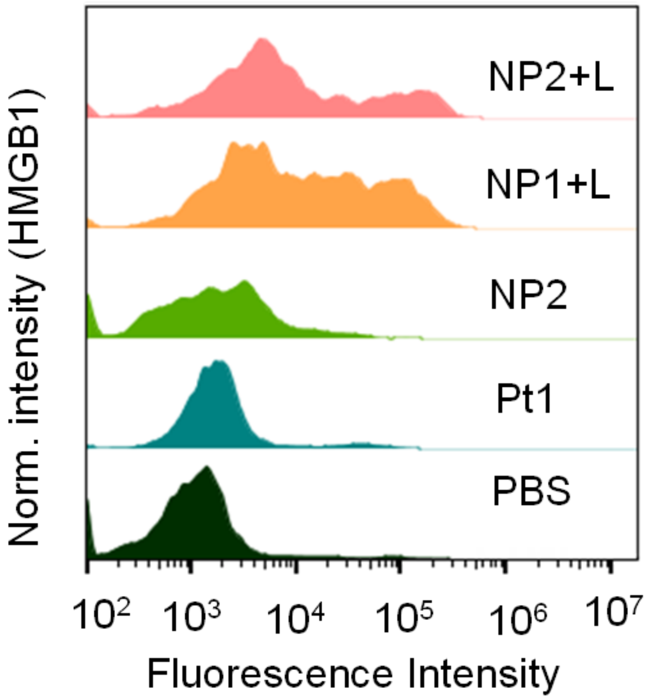

Figure S10. Flow curve for HMGB1 expression in C4-2 cells detected by flow cytometry.

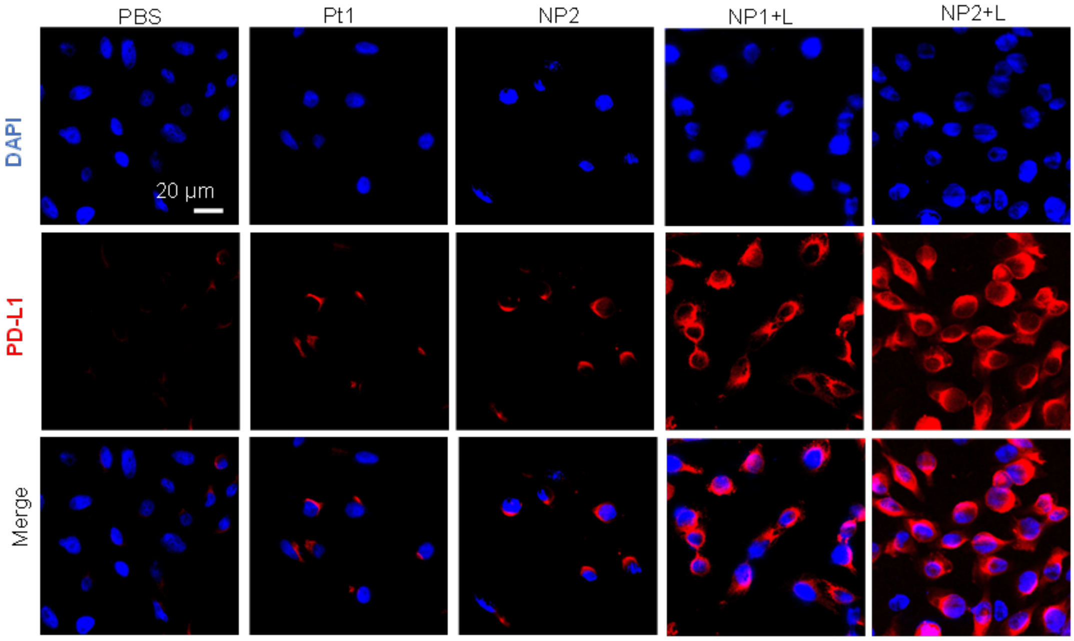

Figure S11. CLSM images of PD-L1 expression on the surface of RM-1 cells after various treatments.

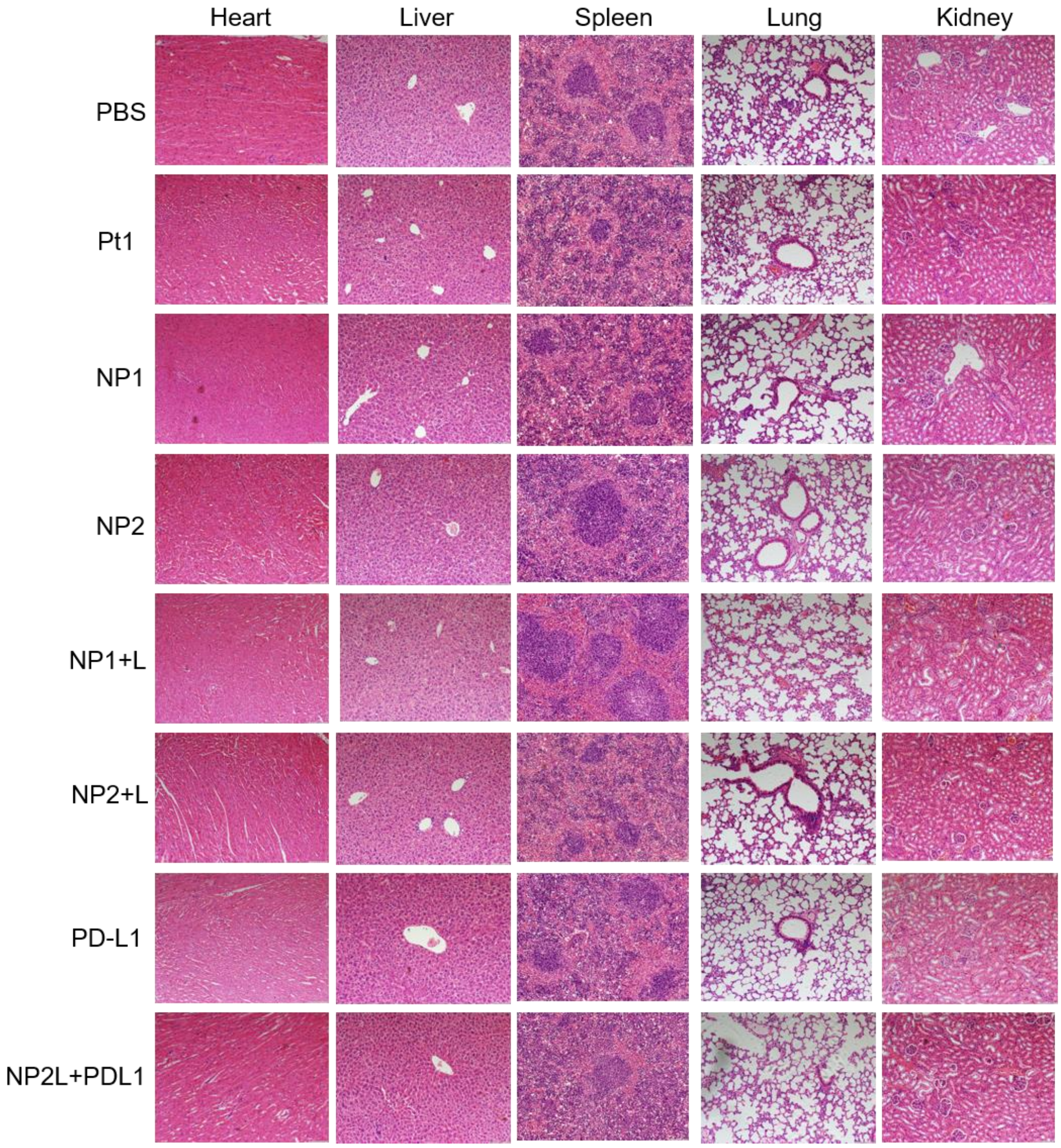

Figure S12. HE staining of heart, liver, spleen, lung and kidney after different drug treatments.

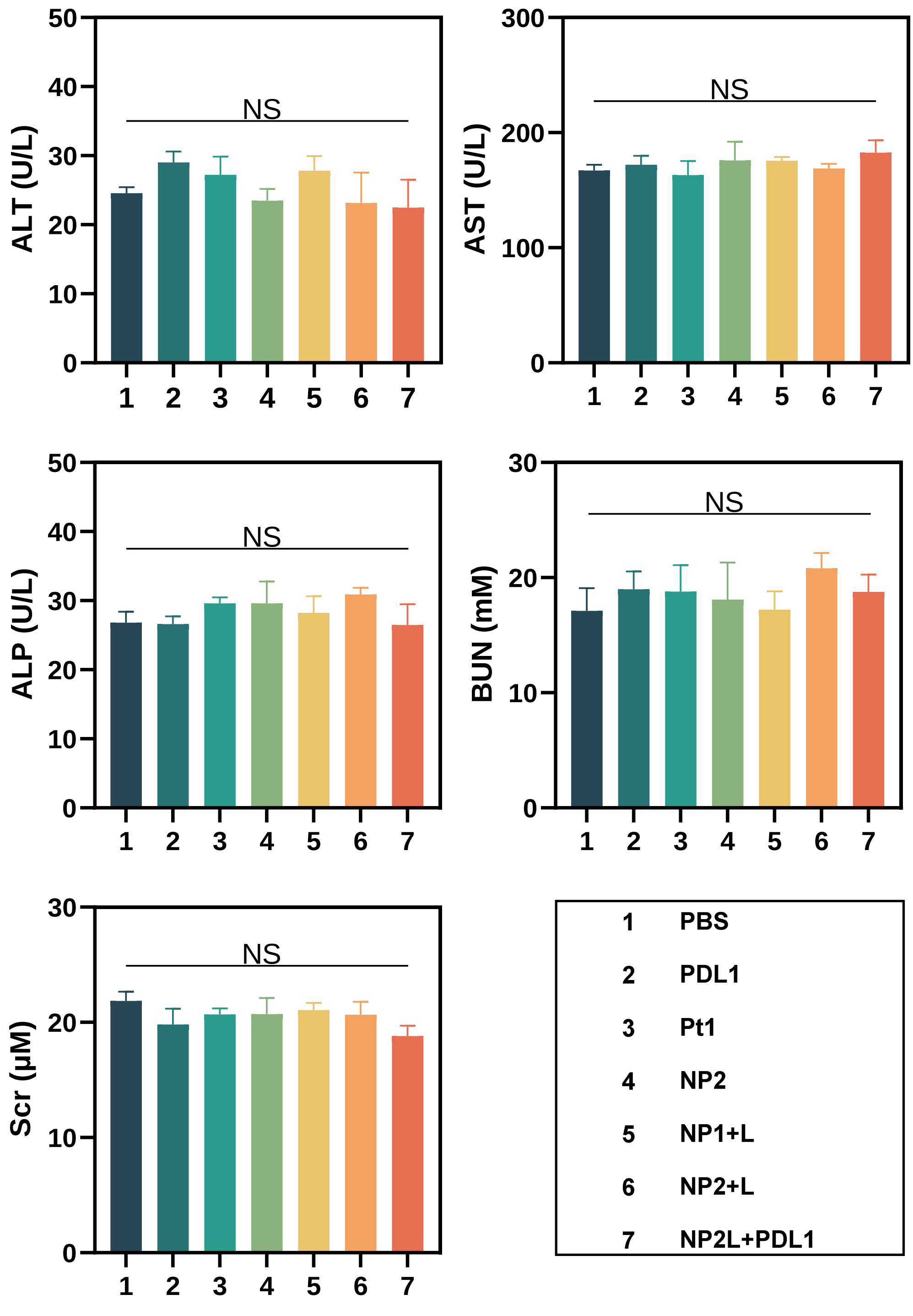

Figure S13. The results of kidney function and liver function after different drug treatments.

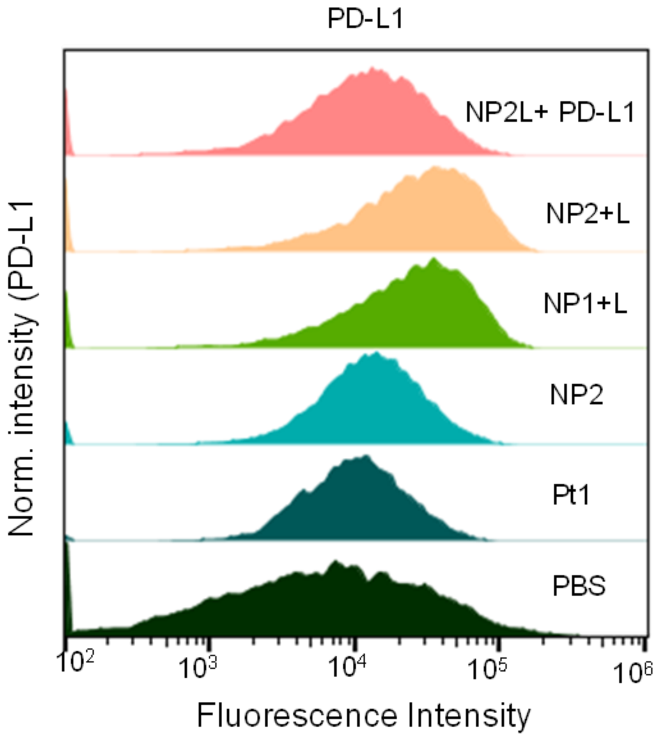

Figure S14. PD-L1 expression detected by flow cytometry.

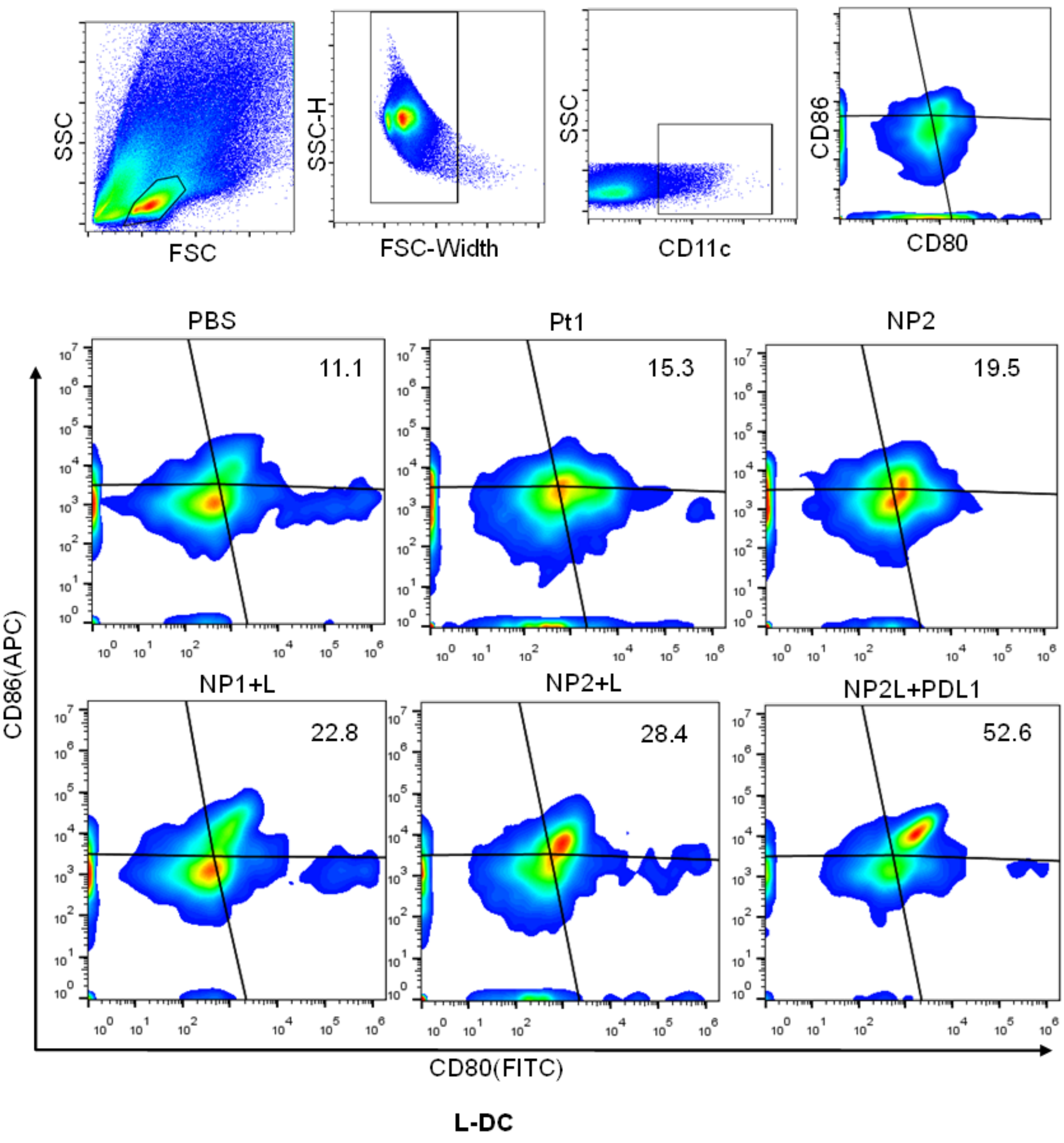

Figure S15. The gating strategy and typical flow cytometer analysis profiles of DCs maturation from each treatment in lymph nodes, wherein matured DCs were denoted as CD1lc^{+} CD80^{+} CD86^{+} cells.

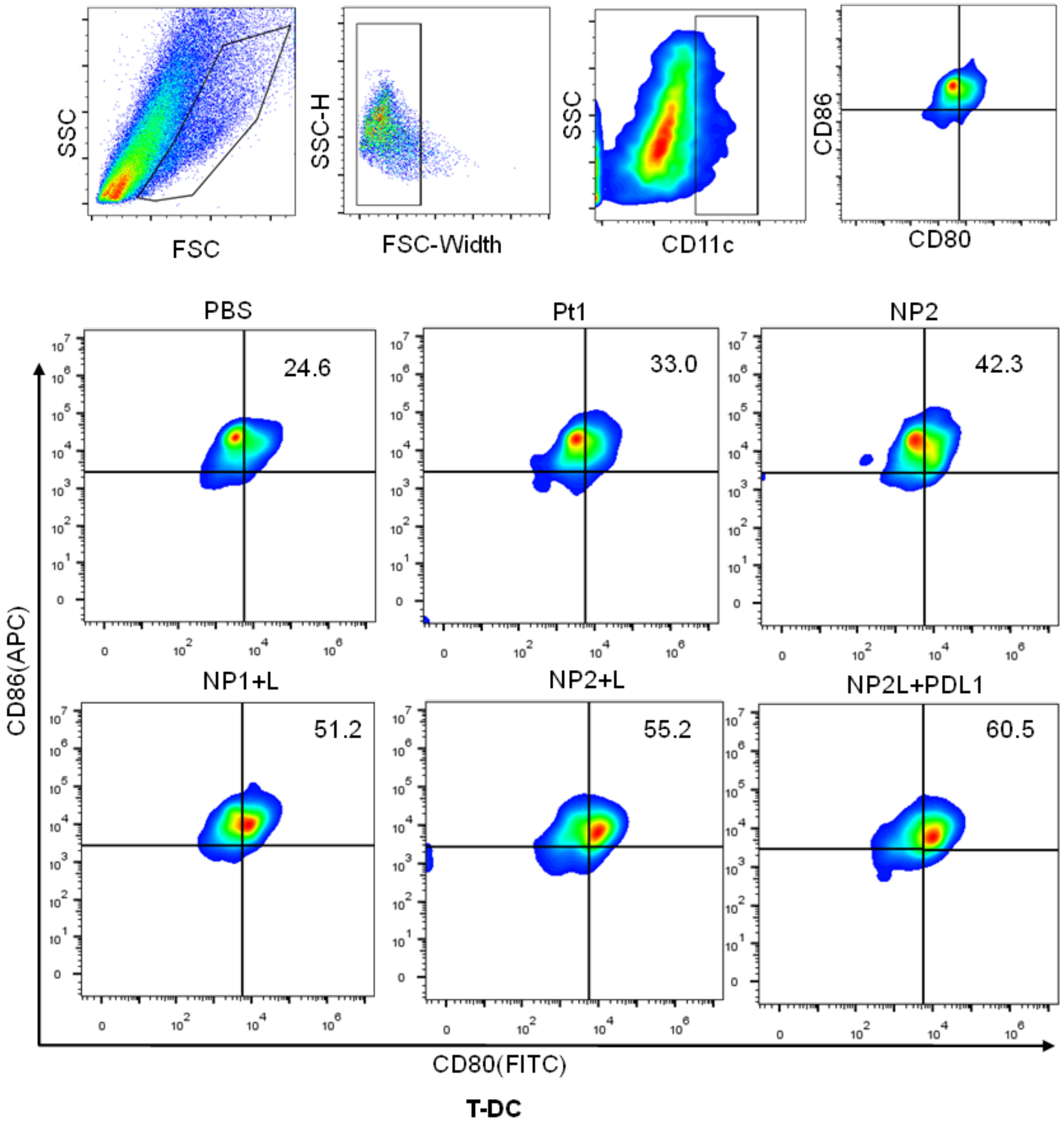

Figure S16. The gating strategy and typical flow cytometer analysis profiles of DCs maturation from each treatment in tumors, wherein matured DCs were denoted as CD1lc^{+} CD80^{+} CD86^{+} cells.

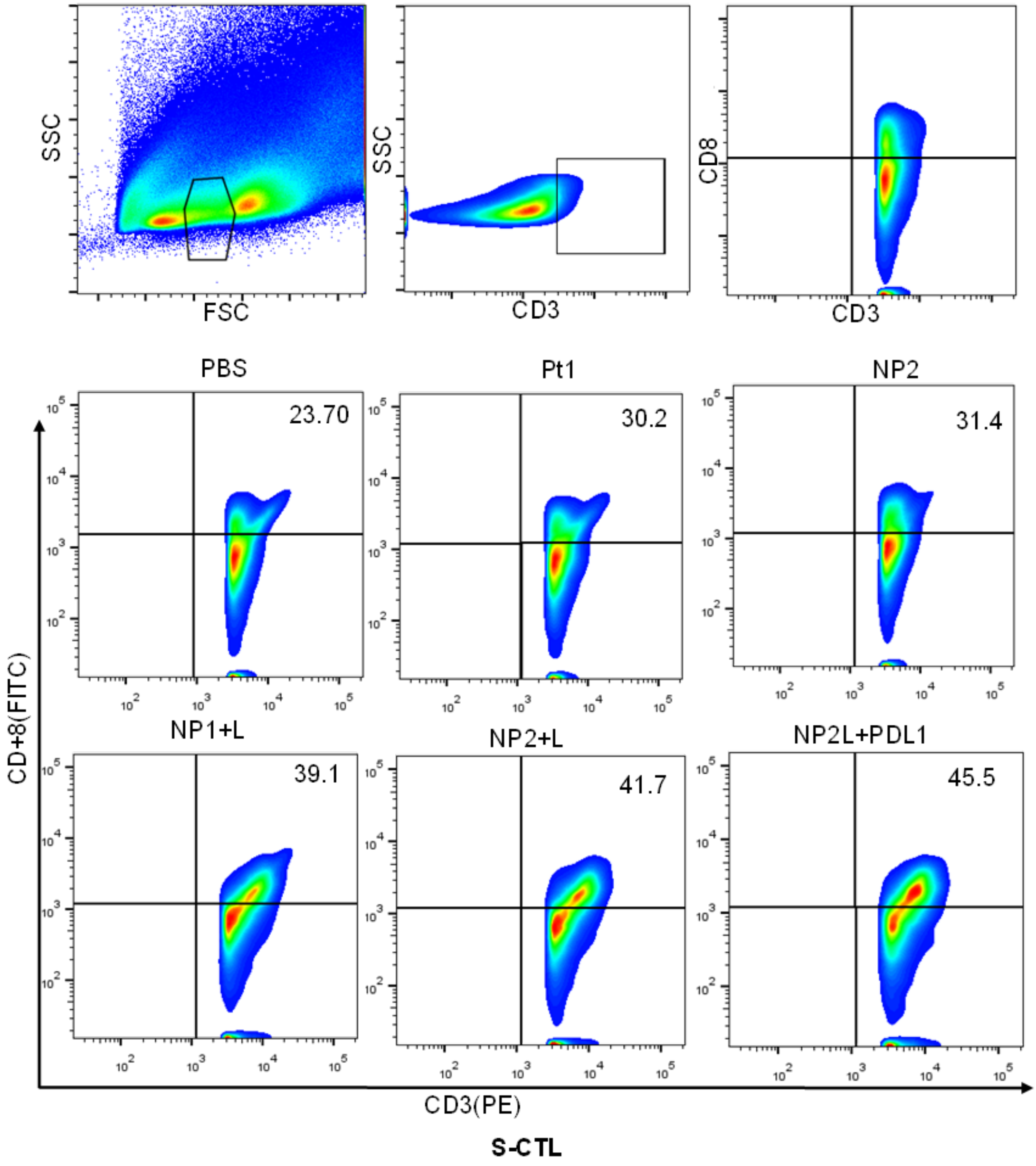

Figure S17. Gating strategies and representative FCM analysis images of CD$^+$ 8 T cells (CD3$^+$CD8$^+$) within spleen tissues after various treatments.

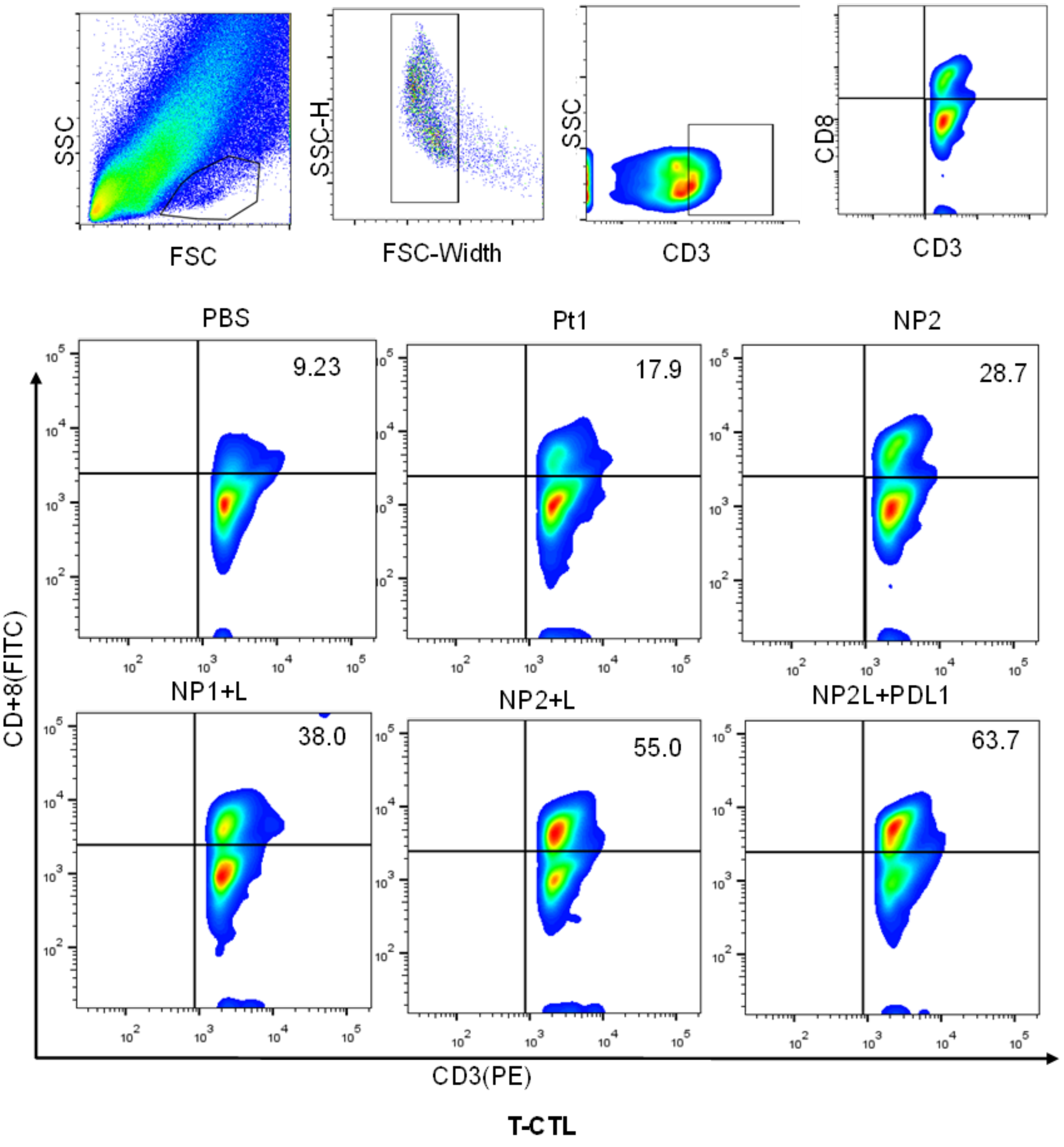

Figure S18. Gating strategies and representative FCM analysis images of CD$^+$ 8 T cells (CD3$^+$CD8$^+$) within tumor tissues after various treatments.

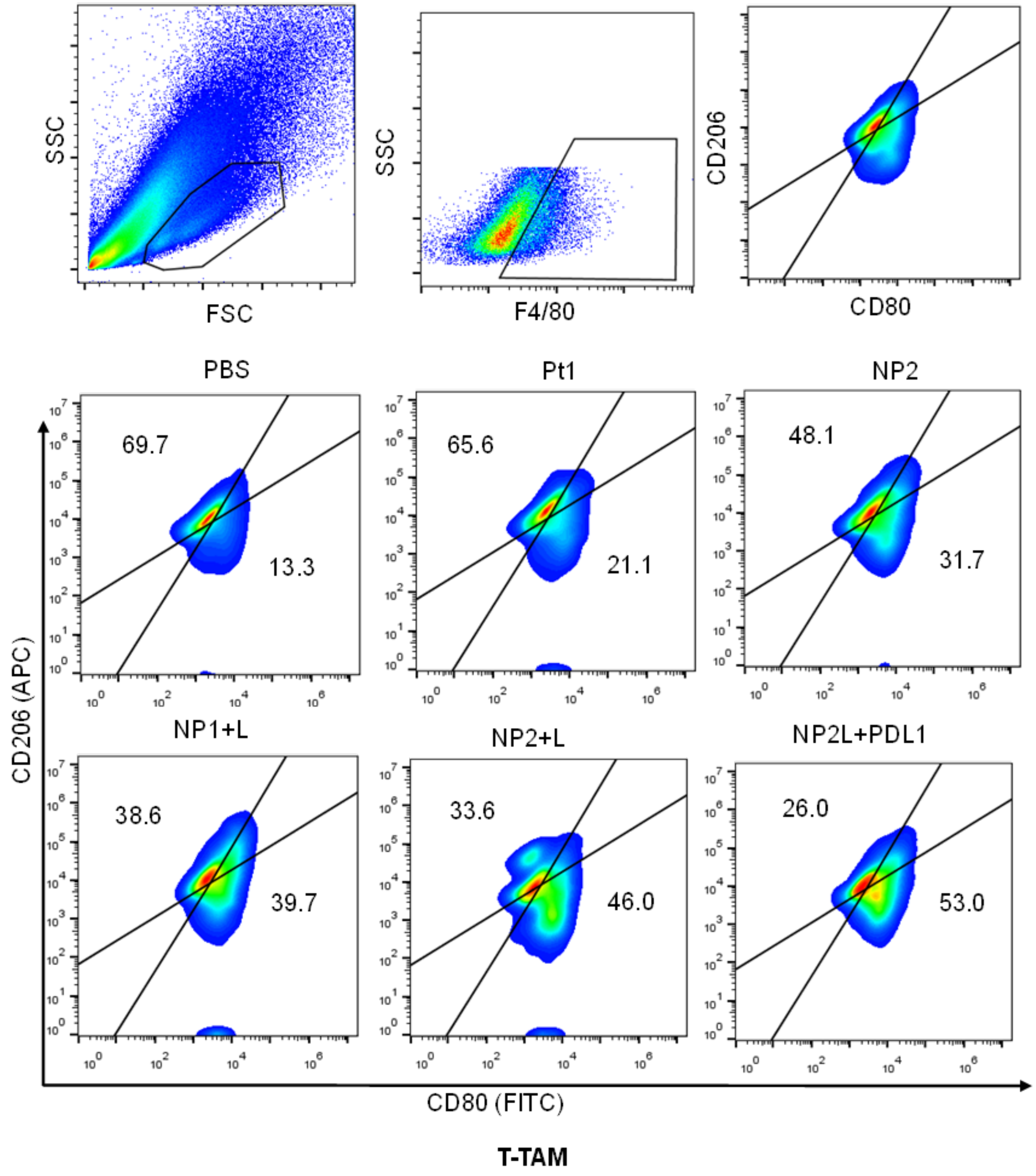

Figure S19. Gating strategies and representative FCM-images of M1 macrophages ($CD80^+CD206^-$) and M2 macrophages ($CD80^-$ $CD206^+$) within tumor tissues after

various treatments.

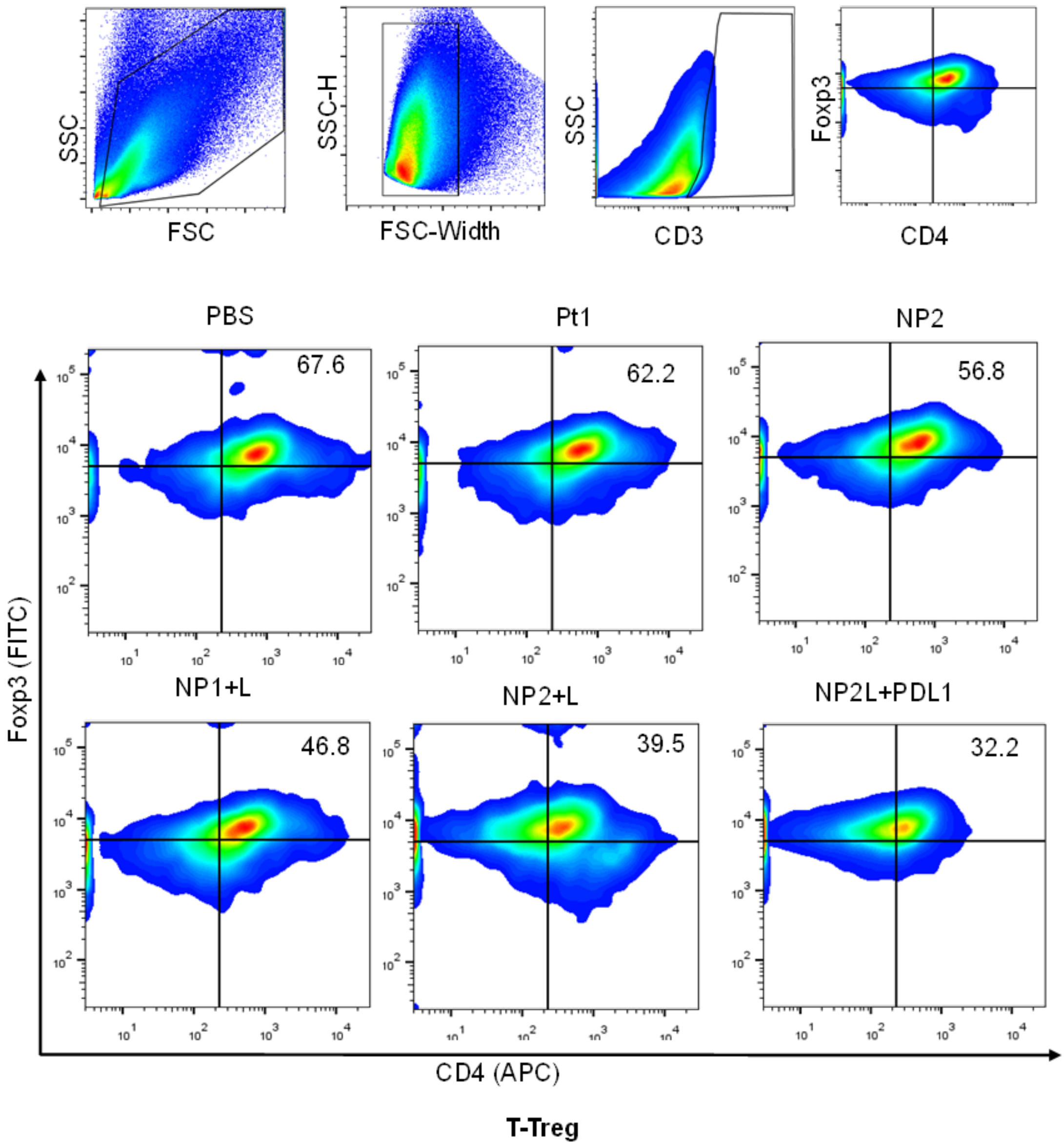

Figure S20. Gating strategies and representative FCM analysis images of Treg cells (CD3^{+}CD4^{+}Poxp3^{+}) within tumor tissues after various treatments.

REFERENCES

[1] X. Zhang, H. Hou, J. Wan, J. Yang, D. Tang, D. Zhao, T. Liu, K. Shang, *Nano Today*, **2023**, 48, 101759.

[2] X. Zhang, J. Wan, F. Mo, D. Tang, H. Xiao, Z. Li, J. Jia,T. Liu, *Adv. Sci.(Weinh)* **2022**, 9, e2201819.
